# Formulations containing co-biotic compounds mediate microbiome function and composition without increased gas production in an *ex vivo* gastrointestinal model

**DOI:** 10.1128/aem.01311-26

**Published:** 2026-10-01

**Authors:** Brooke A. Napier, Bryan D. Merrill, Madeline Krieger, Dirk Gevers, Gregor Reid

**Affiliations:** 1 Seed Health, New York, New York, USA; 2 Lawson Research Institute and Department of Microbiology and Immunology, Western University 6221, London, Ontario, Canada; University of Illinois Urbana-Champaign, Urbana, Illinois, USA

**Keywords:** gut microbiome, deep shot-gun sequencing, prebiotic, tryptophan, short-chain fatty acids, co-biotic, dietary supplements

## Abstract

Dietary supplement formulations that contain co-biotics or compounds that modulate biological processes in both the host and microbiome to confer a health benefit are an emerging strategy to fine-tune both host physiology and gut microbiome function. Here, we evaluated three novel formulations containing co-biotic compounds (DM-02, a multivitamin; AM-02, formulated for energy and focus; and PM-02, formulated to promote sleep) for their effects on human gut microbiome composition and function. The three formulations and untreated control (Unt-Ctrl) were subjected to upper gastrointestinal digestion, after which the digesta were exposed to 24-h simulated colonic fermentation in the validated *ex vivo* systemic intestinal fermentation research (SIFR) technology (*n* = 6 healthy adults). Outcomes included pH, short-chain fatty acid (SCFA) and gas production, ultra-deep metagenomic profiling, and untargeted metabolomics of post-colonic supernatants, each compared to Unt-Ctrl. All three formulations significantly reduced pH (3%–4%) and increased total SCFAs (9%–11%) and acetate (9%–12%), with AM-02 and PM-02 also increasing butyrate by 20% and propionate by 7%–8%, without increasing gas production. Each formulation significantly enriched specific SCFA- and B-vitamin-producing taxa. AM-02 significantly increased the abundance of two pyruvate fermentation to acetate/lactate pathways. PM-02 significantly increased the abundance of two tryptophan biosynthesis pathways, accompanied by an increase in available tryptophan and the abundance of tryptophan-producing microbes. All three formulations increased the availability of microbiome-derived metabolites, indicating microbiome functional modulation by the treatments. These findings support clinical evaluation of these novel formulations as a strategy to enhance microbiome composition and function.

**IMPORTANCE:**

The gut microbiome produces metabolites, including short-chain fatty acids, B vitamins, and tryptophan derivatives, that are critical regulators of host physiology, from energy metabolism and gut barrier integrity to sleep and immune function. While probiotics introduce live microorganisms, and prebiotics selectively feed existing microbes, co-biotics represent a distinct category of compounds that simultaneously modulate host cell biology and microbiome activity. Despite growing interest in co-biotic supplementation, the impact of complete co-biotic-containing formulations on gut microbiome composition and functional output has not been evaluated. Using a validated *ex vivo* gastrointestinal model inoculated with fecal microbiota from six healthy adults, we demonstrate that three supplement formulations containing co-biotic compounds consistently increased production of health-associated metabolites, selectively enriched beneficial microbial taxa, and modulated functional metabolic pathways, all without increasing gas production. These findings establish a mechanistic foundation for the clinical investigation of co-biotic formulations as targeted, tolerable interventions for optimizing gut microbiome function across diverse human populations.

## INTRODUCTION

The gut microbiome supports human health through the production of microbiome-derived metabolites and vitamins that are both utilized locally within the gastrointestinal (GI) system to mediate gut health and systemically to support immune and organ function (1). For example, short-chain fatty acids (SCFAs), specifically acetate and butyrate, are by-products of microbial fermentation of indigestible fibers that decrease colonic pH to support absorption of electrolytes and minerals, promote beneficial bacterial growth, provide a major energy source for colonic epithelial cells, improve gut barrier function, and systemically mediate immune homeostasis and the gut-brain system (2–4). Furthermore, microbiome-derived tryptophan and tryptophan by-products, produced from fermentable carbon and nitrogen sources, support the production of serotonin and melatonin by enterochromaffin cells in the gut epithelium, which influence GI motility and immune balance, and may have systemic impacts on circadian rhythm and sleep (5, 6). Thus, interventions that mediate microbial function and downstream production of microbially derived metabolites can have an important impact on human physiology.

A co-biotic is defined as “a compound that simultaneously modulates biological processes in both the host and microbiome, to confer a health benefit” (7). In comparison to probiotics that introduce live microbes or prebiotics that are substrates selectively utilized by microbes, co-biotics comprise vitamins, minerals, and other compounds known to act on both human and microbial processes in parallel (7). As an example, quercetin is an antioxidant flavonoid found in many fruits and vegetables (e.g., onions, grapes, and cherries) that has been shown to increase the expression of key antioxidant enzymes and directly inhibit inflammatory cytokine production in human cells. Quercetin is associated with reduced blood pressure and systemic inflammation (8–10) and has been shown to induce metabolic changes n the microbiome and potentially shift microbiome community structure in humans (11). Specifically, in a human study, a single dose of quercetin resulted in a significant increase in flavonoid-degrading *Eubacterium ramulus* (11). Other examples of co-biotic compounds include vitamins B1 (thiamine), B2 (riboflavin), and B3 (niacin and nicotinamide), which are essential vitamins (not synthesized by human cells) that act as enzymatic co-factors utilized by both host and microbial cells and have been shown to improve multiple aspects of human health (e.g., GI health, cardiovascular health, metabolism, and vision) and improve the microbiome by increasing beneficial taxa and SCFA production (12–15). Specifically, vitamin B1 is required for human cells to metabolize carbohydrates and branched-chain amino acids (14). Human studies suggest that vitamin B1 is required for the maintenance of butyrate-producing *Ruminococcaceae* taxa (13). Additionally, vitamin B2 is required in human cells for biological redox reactions, energy metabolism, antioxidant pathways, and regulation of inflammation (14). Vitamin B2 has been shown *in vitro* to significantly enrich beneficial taxa (including *Lachnospiraceae* and *Bifidobacterium*) and SCFAs and increase the abundance of *Faecalibacterium prausnitzii* and alpha diversity in humans supplemented with vitamin B2 (12, 14, 15). Finally, vitamin B3 is a potent antioxidant and is required for energy homeostasis in human cells (14), and supplementation has been shown to increase *Bacteroidetes* (14). Therefore, there is growing interest in integrating co-biotic compounds into supplement formulations to support host cell functions while simultaneously fine-tuning microbiome composition and function to synergistically support overall human health.

Three novel supplement formulations (DM-02, AM-02, and PM-02) were engineered with a dual capsule to deliver ingredients (vitamins, minerals, and nutrients) to the upper GI via the outer capsule to target host cell function, as well as co-biotics (quercetin and vitamins B1, B2, and B3) plus a prebiotic (Japanese wasabi root extract) to the colon via the inner capsule to mediate microbiome and host function (12, 13, 15–23). The host-targeted vitamins, minerals, and nutrients within the outer capsule are distinct between the formulations: DM-02 contains a mixture of vitamins and minerals clinically shown to fill nutrient gaps in the diet (e.g., vitamin D and zinc) (24); AM-02 contains American ginseng that has been clinically shown to improve attention and memory (25–27), and PM-02 contains melatonin and ashwagandha that have been clinically shown to improve sleep quality and length (16–19). All three formulations contain the same host- and microbiome-targeted co-biotic compounds within the inner capsule: quercetin and vitamins B1, B2, and B3. Importantly, these three formulations have negligible amounts of carbohydrates within their ingredients.

**Fig 1:**
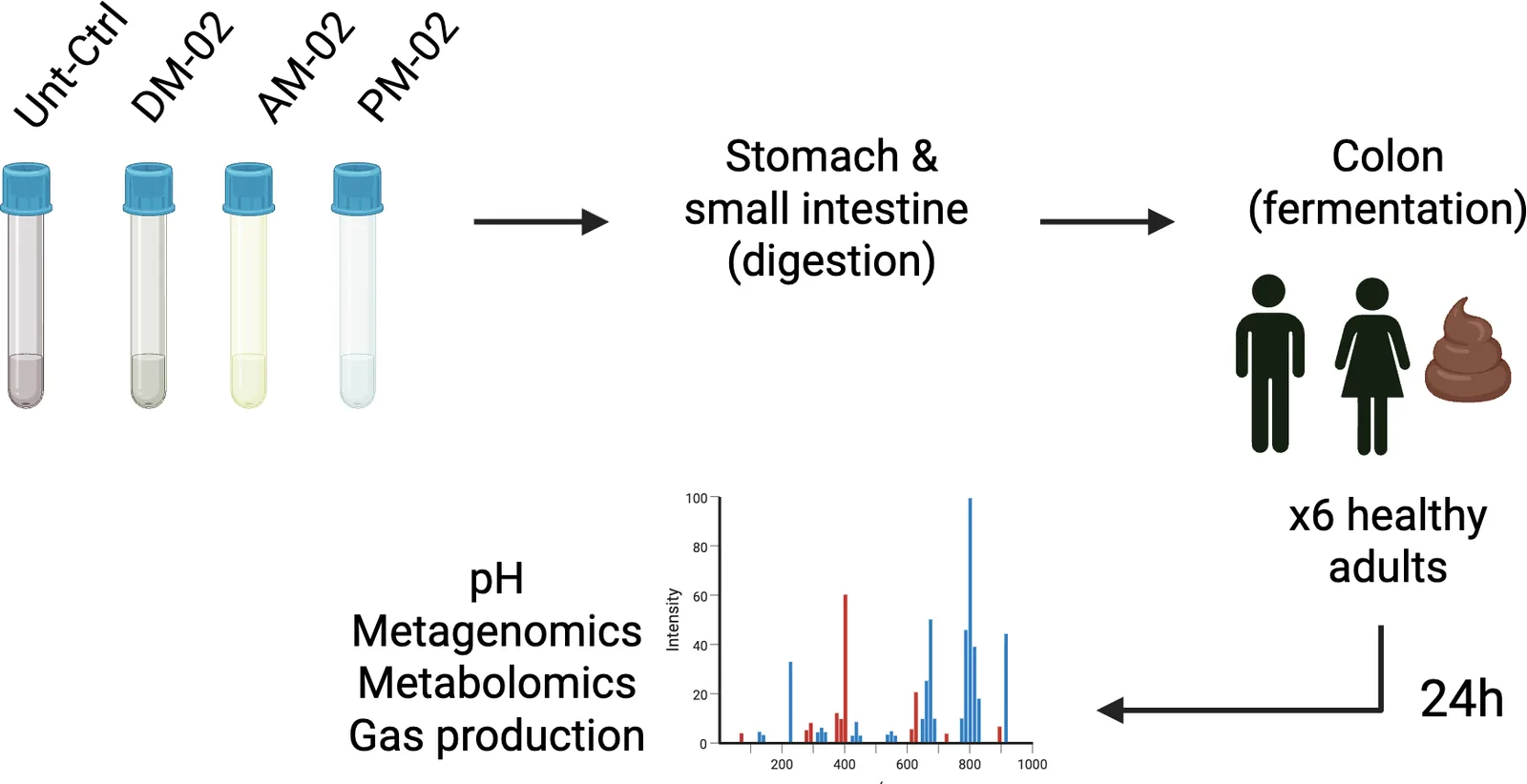
Schematic of the experimental setup to test the impact of three novel formulations containing co-biotics on the function and composition of the human microbiome. Using the validated gastrointestinal (GI) *ex vivo* SIFR technology pipeline, untreated control (Unt-Ctrl) and formulations (DM-02, AM-02, and PM-02) were treated with simulated gastric and small intestine conditions, based on the INFOGEST 2.0 digestion protocol. Next, all samples from the upper GI simulator were combined with healthy human stool (six individual donors) and were subject to a colonic fermentation bioreactor for 24 h. All samples were individually collected and analyzed for key fermentation parameters (pH, SCFA, and gas production), untargeted metabolomics, and sequenced for metagenomic analysis.

Although these co-biotic compounds within these formulations have individually been shown to confer host and microbiome effects, it is not known how each complete formulation containing these co-biotic compounds impacts microbiome function or composition. The aim of this mechanistic preclinical study was to evaluate DM-02, AM-02, and PM-02, engineered with a dual-capsule delivery system, in an *ex vivo* GI model (systemic intestinal fermentation research [SIFR] technology) inoculated with stool from healthy adults (*n* = 6) (Fig. 1). Findings generated using this *ex vivo* technology (within 24 h) have been shown to be predictive of outcomes observed in long-term clinical studies, including changes in microbial composition, gut barrier integrity and immunity, and plasma metabolite levels (28–31). Specifically, the objectives of the study were to determine whether these formulations (i) modulate key fermentative parameters, including pH, SCFA production, and gas generation; (ii) alter microbiome composition and the abundance of taxa predicted to produce key microbiome-derived metabolites and vitamins; and (iii) reshape microbial functional pathways and metabolite profiles in post-colonic supernatants. The findings from this study will provide mechanistic insights that reflect short-term microbiome modulation in a modeled system and will need to be validated in a human study to understand the physiological impact on the host microbiome.

## MATERIALS AND METHODS

### Supplement formulations

DM-02 multivitamin (Seed Health, New York, NY, USA) contains outer-capsule ingredients: 450 mcg vitamin A (retinyl palmitate; H&M Kingdomway, Tustin, CA, USA), 450 mcg vitamin A (β-carotene; Lycored, Branchburg, NJ, USA), 30 mg vitamin C (ascorbic acid; PAT Vitamins, La Verne, CA, USA), 20 mcg vitamin D (cholecalciferol [D3] from algae, AlgeD3, Stauber, Fullerton, CA, USA), 15 mg vitamin E (RRR-α-tocopherol; CellMark, South Norwalk, CT, USA), 120 mcg vitamin K1 (phylloquinone, CellMark), 1.7 mg vitamin B6 (pyridoxal-5-phosphate [P-5-P]; CellMark), 400 mcg vitamin B9 methylfolate (PRO methylfolate [L-5-MTHF Ca]; MAGNAFOLATE, CellMark), 1.2 mcg vitamin B12 (adenosylcobalamin; CellMark), 1.2 mcg vitamin B12 (methylcobalamin; USPL Nutritionals LLC, North Brunswick, NJ, USA), 30 mcg vitamin B7 (d-Biotin; USPL Nutritionals LLC), 5 mg vitamin B5/pantothenic acid (calcium d-pantothenate; CellMark), 150 mcg iodine (potassium iodide; USPL Nutritionals LLC), 5.5 mg zinc (zinc citrate; Stauber, Fullerton, CA, USA), 5.5 mg zinc (zinc lysine glutamate; ZinPro, Eden Prairie, MN, USA), 55 mcg selenium (L-selenomethionine; Stauber), 900 mcg copper (copper bisglycinate chelate; TRAACS, Albion, Layton, UT, USA), 2.3 mg manganese (manganese bisglycinate chelate; TRAACS, Albion), 35 mcg chromium (chromium nicotinate glycinate chelate; TRAACS, Albion), 45 mcg molybdenum (TRAACS, Montvale, NJ, USA; molybdenum glycinate chelate). The inner-capsule ingredients are as follows: 60 mg vitamin C (ascorbic acid; PAT Vitamins), 1.2 mg vitamin B1 (thiamine hydrochloride; CellMark), 1.3 mg vitamin B2 (free riboflavin; CellMark), 5 mg vitamin B3 (niacin/nicotinic acid; CellMark), 11 mg vitamin B3 (niacinamide; CellMark), 30 mg Microbiome-Accessible Phytonutrients (Japanese wasabi [*Wasabia japonica* root extract, 2% isothiocyanates; Compound Solutions, Carlsbad, CA, USA] and Japanese pagoda tree [*Sophora japonica* flower extract, 95% quercetin; CellMark]), and 36 mg Cellular Energy Complex (coenzyme Q10 [ubiquinone, phytosome phospholipid complex; Indena, Milan, Italy], coenzyme PQQ [pyrroloquinoline quinone disodium salt; Centian, Raleigh, NC, USA], spermidine trihydrochloride [CellMark], and vitamin K2 MK-7 [menaquinone-7, a *Bacillus subtilis* var. natto-derived post-biotic; CellMark]).

The AM-02 formulation (Seed Health), designed for energy and focus, contains outer-capsule ingredients: 250 mg Gut-Brain Energy Complex (American ginseng [*Panax quinquefolius* root extract, 10% ginsenosides, Rb1, Re, Rc, Rd, Rb2, and Rg1; Cereboost, Givaudan, Vernier, Switzerland], methylliberine [SoWell, Compound Solutions], and theacrine [TeaCrine, Compound Solutions]). Inner-capsule ingredients comprise 100 mg gamma-aminobutyric acid (GABA, a *Lactobacillus brevis*-derived post-biotic; CellMark), 30 mg Microbiome Accessible Phytonutrients (Japanese wasabi [*Wasabia japonica* root extract, 2% isothiocyanates; Compound Solutions] and Japanese pagoda tree [*Sophora japonica* flower extract, 95% quercetin; CellMark]), 10 mg Bioenergetic Cellular Complex (coenzyme Q10 [ubiquinone, phytosome phospholipid complex; Indena], coenzyme PQQ [pyrroloquinoline quinone disodium salt; Centian], vitamin K2 MK-7 [menaquinone-7, a *B. subtilis* var. natto-derived post-biotic; CellMark]), 1.2 mg vitamin B1 (thiamine hydrochloride; CellMark), 1.3 mg vitamin B2 (free riboflavin; CellMark), 5 mg vitamin B3 (niacin/nicotinic acid; CellMark), 0.4 mg vitamin B9 methylfolate (PRO methylfolate [L-5-MTHF Ca]; MAGNAFOLATE, CellMark), 1.2 mcg vitamin B12 (adenosylcobalamin, CellMark), and 1.2 mcg vitamin B12 (methylcobalamin; USPL Nutritionals LLC).

The PM-02 formulation (Seed Health) designed for sleep and restoration contains the following outer-capsule ingredients: 500 mcg Extended Release Biphasic Melatonin (melatonin [rapid release; USPL Nutritionals LLC] and melatonin [MicroActive, MayPro, Purchase, NY, USA]), 140 mg Restorative Sleep Complex (ashwagandha [*Withania somnifera* root and leaf extract, 35% withanolide glycosides; Shoden, Arjuna Naturals, Irving, Texas, USA] and coenzyme PQQ [pyrroloquinoline quinone disodium salt; Centian]), and 100 mg gamma-aminobutyric acid (GABA, a *L. brevis*-derived post-biotic; CellMark). The inner-capsule ingredients are as follows: 100 mg gamma-aminobutyric acid (GABA, a *L. brevis*-derived post-biotic; CellMark), 30 mg Microbiome-Accessible Phytonutrients (Japanese wasabi [*Wasabia japonica* root extract, 2% isothiocyanates; Compound Solutions] and Japanese pagoda tree [*Sophora japonica* flower extract, 95% quercetin; CellMark]), 10 mg Cellular Renewal Complex (pterostilbene [trans-pterostilbene, CellMark] and spermidine trihydrochloride [CellMark]), 1.2 mg vitamin B1 (thiamine hydrochloride; CellMark), 1.3 mg vitamin B2 (free riboflavin; CellMark), and 5 mg vitamin B3 (niacin/nicotinic acid; CellMark).

### Fecal donor selection

The selection criteria for the human adult donors were as follows: age 25–50 years, a BMI of 18.5–25, and a Bristol stool score of 3–4. The exclusion criteria included antibiotic use in the past 3 months; prebiotic, probiotic, or dietary supplement usage (including botanical extracts and oils) within 3 months of the study; smoking; recreational and chronic prescription drug usage (including cannabis); alcohol consumption >3 units/day; chronic gastrointestinal (GI) issues (constipation or diarrhea); and diagnosed disorders (cancer, GI disorders such as IBD/Crohn’s/ulcers, or clinically diagnosed mental health issues) (Table S1).

### *Ex vivo* GI model

The *ex vivo* SIFR technology involves a bioreactor-based technology that maintains the composition and functionality of the original donor microbiota, including difficult-to-culture species, thereby enabling clinically relevant evaluation of microbiome responses to interventions. *Ex vivo* SIFR experiments were performed as previously described (28–30). First, one capsule of each formulation, or a digestion blank serving as an untreated control (Unt-Ctrl), was subjected to oral, gastric, and small intestinal digestion according to the INFOGEST 2.0 method (32). First, one capsule of each formulation, or a digestion blank serving as the untreated control (Unt-Ctrl), was subjected to a modified INFOGEST 2.0 gastric and small intestinal digestion procedure (32). To ensure compatibility with the subsequent colonic incubations, oxygen was removed throughout the digestion procedure. Based on visual observation of rapid capsule dissolution, the gastric incubation phase was shortened to 30 min to better reflect the progressive gastric emptying of the released capsule contents *in vivo*. Furthermore, following small intestinal digestion, the resulting suspensions were directly introduced into the colonic incubations. As the formulations contained no digestible macronutrients, such as starch, protein, or fat, no dialysis-based simulation of small intestinal absorption was applied. For subsequent colonic simulations (Fig. 1), individual bioreactors were processed in parallel in a bioreactor management device (Cryptobiotix, Ghent, Belgium). Each bioreactor contained 5 mL of nutritional medium-fecal inoculum blend plus upper GI suspension and was then sealed individually before being rendered anaerobic. Blend M0017 was used for preparation of the nutritional medium (Cryptobiotix, Ghent, Belgium). After preparation, bioreactors were incubated under continuous agitation (140 rpm) at 37°C (MaxQ 6000, Thermo Scientific, Thermo Fisher Scientific, Merelbeke, Belgium). Upon total gas pressure measurement in the headspace, liquid samples were collected for subsequent analysis.

### Key fermentation parameters

At 24 h, SCFAs (acetate, propionate, and butyrate) were determined via gas chromatography with flame ionization detection (Trace 1300, Thermo Fisher Scientific, Merelbeke, Belgium), upon diethyl ether extraction, as previously described (30). The pH was measured via a fluorescence assay (Sigma-Aldrich, C3411-1MG), and the accumulation of gases in the headspace was determined by a needle connected to a pressure meter, as previously described (28). Raw data for all six donors can be found in Table S2.

### Bacterial composition

To determine total bacterial counts at 24 h, samples were diluted in anaerobic phosphate-buffered saline (PBS), followed by cell staining with SYTO 16 at a final concentration of 1 μM, and counted via a BD Novocyte Quanteon (Agilent, Santa Clara, CA, USA). Data were analyzed using NovoExpress, version 1.6.2.

Next, DNA was extracted via the SPINeasy DNA Kit for Soil (MP Biomedicals, Eschwege, Germany), according to the manufacturer’s instructions. Libraries were prepared using the Watchmaker DNA Library Prep Kit, with genomic DNA being fragmented using a master mix of Watchmaker Frag/AT Buffer and Frag/AT Enzyme Mix (Watchmaker Genomics, Boulder, CO, USA). IDT xGen UDI Primers and IDT Stubby Adapters were added, and DNA libraries were constructed through seven cycles of PCR, after which they were purified utilizing CleanNGS magnetic beads (CleanNA, Waddinxveen, the Netherlands) and eluted into nuclease-free water. Subsequently, library quantification was done using the Qubit Fluorometer dsDNA HS Assay Kit (Thermo Fisher Scientific, Merelbeke, Belgium). Libraries were circularized following the Element Adept library compatibility workflow and then sequenced on the Element AVITI platform using the AVITI 2 × 150 Cloudbreak sequencing kit (Element Biosciences, San Diego, CA, USA).

### Taxonomic and metagenomics analysis

Sequenced reads were first processed via shi7_trimmer v0.92f to remove regions containing sequencer adapters or low-quality bases. Reads that mapped against the human genome via canola v0.86 were discarded. Following these QC steps, the median sample had 45 million reads (~13.5 Gbp). These remaining reads were profiled against a database containing each species-representative genome in GTDB release 226 (which are dereplicated at ~95% ANI) using xtree v2.00b. Xtree decomposes input genomes into k-mers, using k-mers unique to each input genome for profiling. The xtree database was built using a k-mer size of 29 and compression set to --comp 2. Samples were profiled using parameters --redistribute and --half-forage to enable maximum-likelihood redistribution of reads. Species with ≥1% of the genome’s unique k-mers contained within reads in a sample were passed on to downstream analysis.

The relative abundance of each taxon was computed by dividing the coverage depth by the summed coverage depth for all taxa in a sample. Absolute abundance was computed by multiplying the relative abundance by the calculated cells per mL measured in that sample. The log10 absolute abundance of each taxon was modeled in response to input condition, with fecal donor as a random effect in R (version 4.3.3) using limma (version 3.58.1) and the dream function from the variancePartition package (version 1.32.5).

For functional characterization of each species present in the data set, all genomes in GTDB R226 belonging to that species were downloaded. Metadata from the Prokaryote Genome Annotation Pipeline (PGAP) v18 was screened to identify Hidden Markov Models (HMMs) associated with metabolic pathways of interest, with a focus on terminal enzymes producing metabolites of interest; for example, genomes containing the butyrate kinase (buk, PGAP TIGR02707.1) were marked as butyrate producers. hmmsearch v3.4 (with options -Z 18057 --cut_tc) was used to determine the presence of each terminal enzyme PGAP in each genome. Within each species, the proportion of genomes containing a given terminal enzyme was determined. To indicate whether a species is a “producer” or “non-producer,” two prevalence thresholds for terminal enzyme presence were used: lenient (2% or 50% of the genomes in the species, helpful for species with very few genomes) and stringent (75% of genomes in the species). A genus was labeled as a “producer” if one species and two-member genomes were labeled as “producers.”

### Microbiome diversity

Relative abundance values from taxonomic profiling were used to conduct alpha- and beta-diversity analysis. For each sample, alpha-diversity was computed in R using the vegan package (version 2.7-3) on the Shannon diversity index. Principal coordinate analysis (PCoA) plots were constructed from a pairwise Bray-Curtis distance matrix computed using the vegan package (version 2.7-3) in R. Points were colored by test condition and shaped by donor. ggplot2 was used to create both alpha- and beta-diversity plots.

### HUMAnN3 functional analysis

HUMAnN3 analysis was conducted on human-depleted concatenated FASTQ files containing forward and reverse reads for each sample. The workflow was run from a conda environment containing HUMAnN3 (version 3.9) and MetaPhlAn (version 4.1.1) using the ChocoPhlAn (version v201901_v31) and Uniref90 (version 201901b full) databases. Pathway abundance tables were joined using the humann_join_tables command. Community-level counts from these tables were transformed into relative abundance and analyzed as described above in Taxonomic and metagenomics analysis. Bar plots were constructed with ggplot2 to show pathways with significant Benjamini-Hochberg (BH) adjusted *P*-values (*P* ≤ 0.05). Bar height represents the pathway-level log10 fold change estimate. Bar fill indicates enrichment direction. Pathways were ranked by descending log10-fold change estimate and then by ascending adjusted *P*-value. Pathways labeled “unmapped” or “unintegrated” were excluded.

Pathways of interest were further analyzed to detect shifts in the relative contribution by individual bacterial species resulting from formulation treatment. The species-level data from the pathway of interest were subsetted from the HUMAnN pathway results table and transformed into relative abundance per sample and treatment group. These values were then log10-transformed. A delta matrix was computed by subtracting the value of the untreated control from the within-donor treatment values (DM-02, AM-02, or PM-02) to show changes in relative species-level contributions for the pathway after treatment. A two-sided *t*-test was then used to determine if the delta matrix for a given species was significantly different from zero. Species with *P*-values ≤ 0.05 were retained. We used both the xtree output (described above) and the output of MetaPhlAn, which is run by default in the HUMAnN3 pipeline, to ensure that none of the bacterial species that were significantly differentially contributing to pathway enrichment were themselves differentially enriched. Unassigned bacterial taxa were excluded from plotting.

### Untargeted metabolomics analysis

Semi-polar metabolites were analyzed using liquid chromatography coupled to tandem mass spectrometry. Chromatographic separation was performed on a Vanquish LC system connected to an Orbitrap Exploris 240 mass spectrometer (Thermo Fisher Scientific, Waltham, MA, USA), using an adapted UHPLC method based on Doneanu et al. (33). Electrospray ionization was applied, and data were acquired in polarity-switching mode to capture both positive and negative ion signals.

Untargeted data processing was carried out with Compound Discoverer 3.3 (Thermo Fisher Scientific, Waltham, MA, USA) and Skyline 25.1 (MacCoss Lab Software, Seattle, WA, USA) for peak detection and feature grouping. Candidate features were subsequently annotated and curated using an in-house pipeline developed in MATLAB (MathWorks, Natick, MA, USA). Downstream analyses were restricted to metabolites with high-confidence annotations, supported by retention time matching to in-house authentic standards, accurate mass within 3 ppm, and, when available, MS/MS spectral information. To enrich metabolites generated during microbial incubation, statistical analyses focused on compounds whose signal increased between baseline (0 h, Unt-Ctrl) and 24 h in at least one treatment condition and in at least four of six subjects within an age group. Analytical reproducibility was monitored by injecting a pooled quality-control sample, prepared from all study samples, every six injections. Across the metabolites retained for analysis, the average coefficient of variation was 13.2%

Volcano plots for the full results of untargeted metabolomics were constructed with ggplot2 to show a log2 fold change estimate (log2fc_est) and a Benjamini-Hochberg adjusted *P*-value. The vertical axis shows -log10(adjusted *P*-value). Metabolites with an adjusted *P*-value ≤ 0.05 and positive or negative log2-fold change were classified as up- or down-regulated relative to the Unt-Ctrl. Horizontal reference lines were drawn at adjusted *P*-value = 0.05; vertical lines at log2-fold change 0 and at ±1 served as visual guides.

### Statistical analysis

Significance was assessed between arms using linear mixed-effects modeling (LME). The analysis was conducted using the dream function within the variancePartition package (version 1.32.5), which is an extension of the limma framework (version 3.58.1) to handle repeated measures designs. *P*-values for all tests were adjusted for multiple comparisons using the Benjamini-Hochberg method to control the False Discovery Rate (FDR).

### Correlation analysis

The HUMAnN3 pathway abundance table and the metabolite abundance tables were both scaled to relative abundance values. Delta matrices were then computed for each treatment condition (DM-02, AM-02, and PM-02) to describe the treatment change from Unt-Ctrl for each pathway and metabolite. Pathways labeled “unmapped” or “unintegrated” were excluded from further analysis. We then computed all pairwise associations between every metabolite delta vector and every pathway delta vector across those matched samples using Pearson correlation (base R).

## RESULTS

### SCFA and gas production

**Fig 2:**
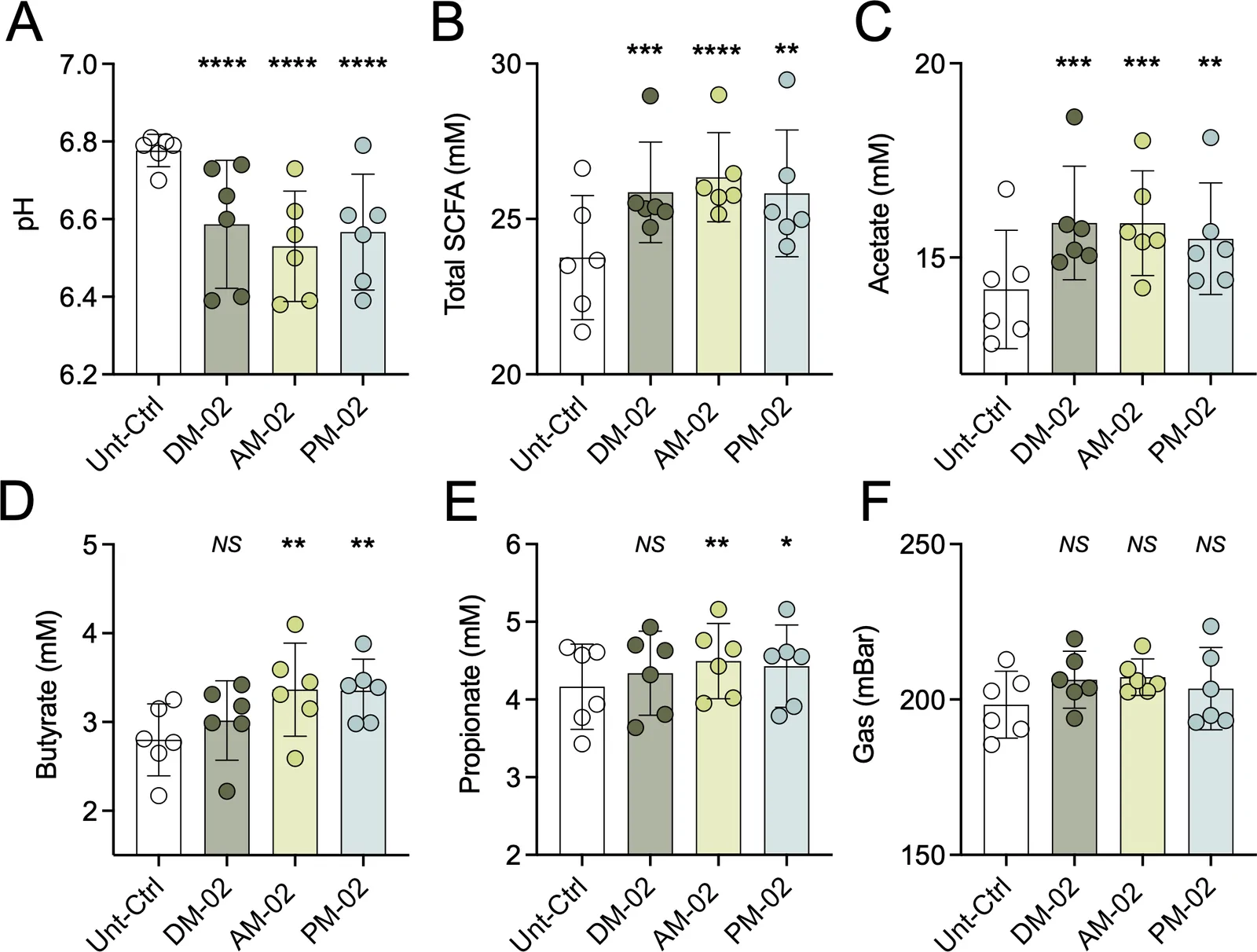
Formulations containing co-biotics decrease luminal pH and increase SCFA production without increasing gas production. The three formulations (DM-02, AM-02, and PM-02) and Unt-Ctrl were subject to simulated digestion and then combined with healthy human stool from six individuals and incubated with colonic simulation for 24 h. (**A**) pH, (**B**) total SCFA (mM), (**C**) acetate (mM), (**D**) butyrate (mM), (**E**) propionate (mM), and (**F**) gas (mBar) production were measured. Significance was measured between arms using linear mixed-effects modeling (LME). *P*-values for all tests were adjusted for multiple comparisons using the Benjamini-Hochberg method. *, *P* < 0.05; **, *P* < 0.01; ***, *P* < 0.001; ****, *P* < 0.001.

Considering what is known about the co-biotic compounds quercetin, vitamin B1, B2, and B3 modulating the production of SCFAs, specifically acetate and butyrate, we hypothesize that key fermentation parameters including colonic pH, SCFA production, and gas production will be regulated by these three formulations. We found that all three formulations containing co-biotics significantly decreased colonic pH at 24 h compared to the untreated control (Unt-Ctrl): DM-02 decreased by 3% (*P* < 0.0001), AM-02 decreased by 4% (*P* < 0.0001), and PM-02 decreased by 4% (*P* < 0.0001) (Fig. 2A; Fig. S1A). The decrease in colonic pH coincided with an increase in SCFA production. DM-02 increased total SCFAs by 9% (*P* < 0.001) and acetate production by 12% (*P* < 0.001) (Fig. 2B and C; Fig. S1B and C). AM-02 increased total SCFAs by 11% (*P* < 0.0001), acetate production by 12% (*P* < 0.001), butyrate production by 20% (*P* < 0.01), and propionate production by 8% (*P* < 0.01) (Fig. 2B through E; Fig. S1B through E). PM-02 increased total SCFAs by 9% (*P* < 0.01), acetate production by 9% (*P* < 0.01), butyrate production by 20% (*P* < 0.01), and propionate production by 7% (*P* < 0.05) (Fig. 2B through E; Fig. S1B through E). These data indicate that the three formulations confer a prebiotic effect, promoting microbial species to selectively utilize the model substrate to increase the production of SCFAs, known to confer multiple health benefits in humans (4).

Often, prebiotic fermentation by microbes in the colon leads to the co-production of SCFAs and gases, such as hydrogen and carbon dioxide (34). Widely used fiber-based prebiotics, such as inulin, have been shown to simultaneously increase SCFA and induce significant gas production (35). The three formulations containing co-biotics did not increase gas production (Fig. 2F; Fig. S1F).

In summary, these three formulations decreased pH and increased SCFA production, without increasing gas production in this model system. Additionally, although these findings are not causative, it is likely that the associated decrease in pH is due to the significant increase in SCFA production induced by these formulations.

### Microbiome profile

Overall, alpha- (Shannon Diversity) and beta-diversity (Bray-Curtis) were not significantly different between Unt-Ctrl and all three formulation treatments (DM-02, AM-02, and PM-02; Fig. S2A and B). The donor fecal sample was the strongest predictor of beta-diversity grouping (Fig. S2B).

**Fig 3:**
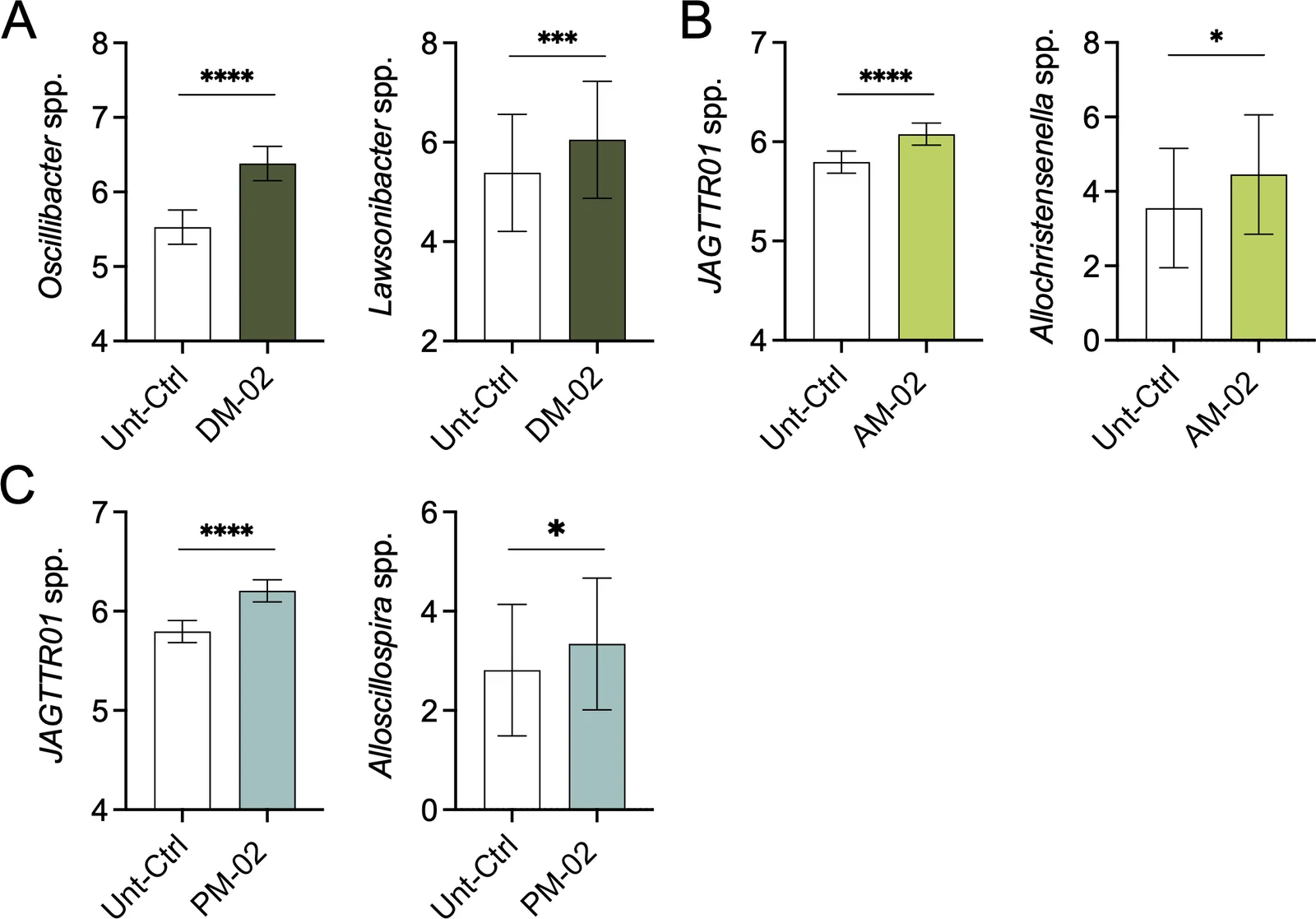
Formulations containing co-biotics significantly increase the absolute abundance of microbes that produce SCFAs. As previously described, samples were collected after 24 h of colonic fermentation with fecal microbiomes from six individual healthy adults. Log2 absolute abundance of specific species within colonic supernatant from the (**A**) DM-02, (**B**) AM-02, or (**C**) PM-02 formulation treatments was quantified through taxonomic profiling of the sample metagenomes. Error bars represent the standard deviation within the treatment. Significance was measured between arms using LME. *P*-values for all tests were adjusted for multiple comparisons using the Benjamini-Hochberg method. *, *P* < 0.05; **, *P* < 0.01; ***, *P* < 0.001; ****, *P* < 0.001.

The absolute abundance of individual species significantly varied between formulation treatments and Unt-Ctrl at 24 h, indicating that shifts in specific metabolite- or vitamin-producing microbes were altered via the formulations with co-biotic compounds. In alignment with SCFA production results, DM-02 significantly increased the absolute abundance of acetate- and butyrate-producing species from the genera *Oscillibacter* by 618% (*P* < 0.0001) and *Lawsonibacter* by 363% (*P* < 0.001) compared to Unt-Ctrl (Fig. 3A). Using this analysis, these species of *Oscillibacter* and *Lawsonibacter* increased by DM-02 treatment were also identified as producers of vitamin B1 (thiamine) and vitamin B2 (riboflavin), both of which act as cofactors for microbial metabolism or are used as nutrients for host cells in the colon (14, 36). Furthermore, the species of *Lawsonibacter* that was increased by DM-02 was labeled as a producer of vitamin B7 (biotin). Importantly, in accordance with gas production results, DM-02 decreased the absolute abundance of a gas-producing microbe, *Methanocatella smithii*, by 80%, compared to Unt-Ctrl (*P* < 0.05) (Fig. S3A).

AM-02 treatment significantly increased the absolute abundance of acetate- and butyrate-producing species from the genus JAGTTR01 (92% increase, *P* < 0.0001) and *Allochristensenella* (700% increase, *P* < 0.05), compared to Unt-Ctrl (Fig. 3B). This species of JAGTTR01 was also labeled as a producer of vitamin B1, vitamin B2, and vitamin B12. Interestingly, PM-02 increased the absolute abundance of the same species from the genus JAGTTR01 (158% increase, *P* < 0.0001; Fig. 3C), suggesting that the co-biotics in the three formulations may have a similar impact on this SCFA- and vitamin B-producing species.

Furthermore, PM-02 significantly increased the absolute abundance of another acetate- and butyrate-producing species from the genus *Alloscillospira* (236% increase, *P* < 0.05), compared to Unt-Ctrl (Fig. 3C). This species of *Alloscillospira* is also a producer of vitamin B1.

### Microbiome functional capacity

**Fig 4:**
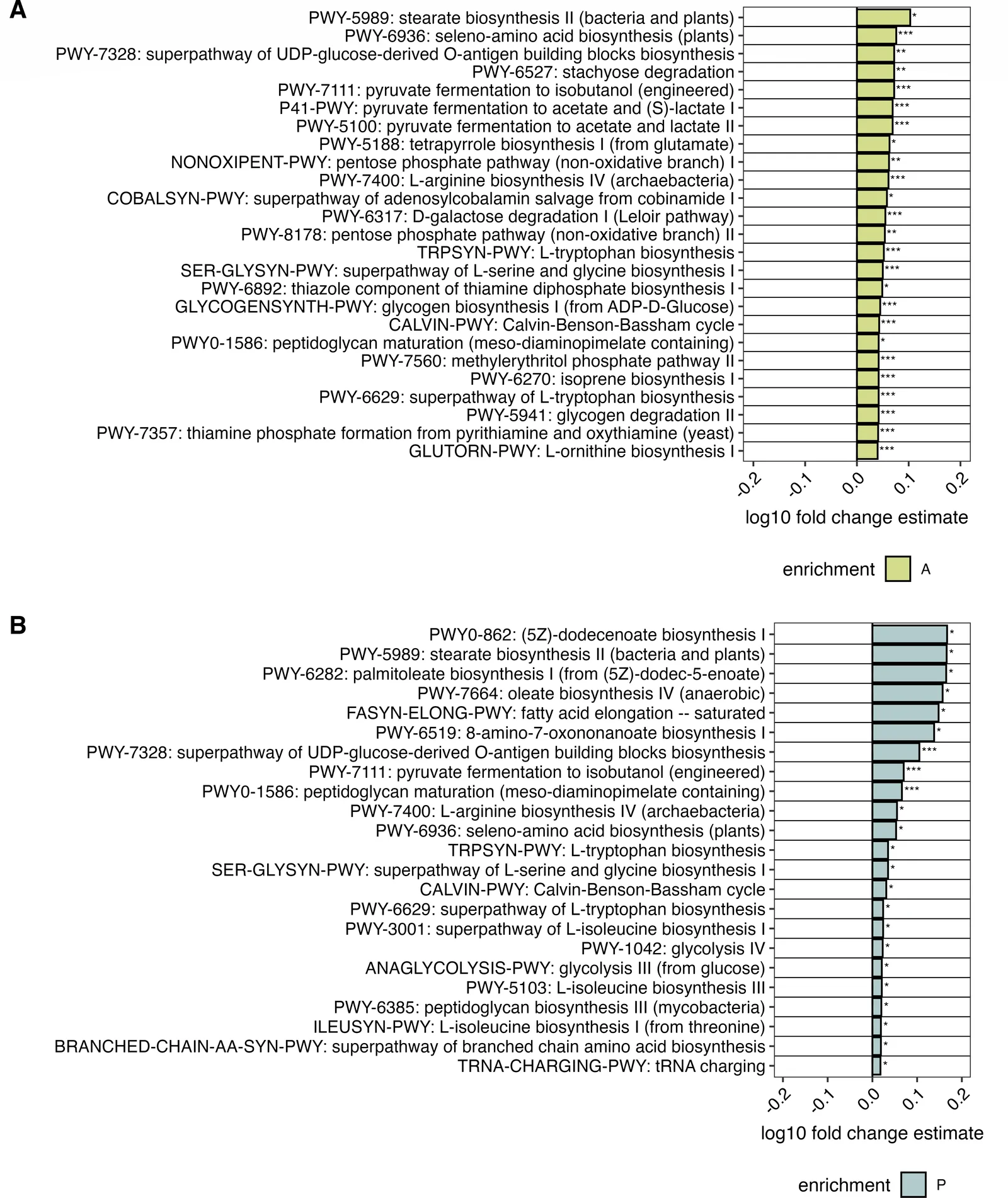
AM-02 and PM-02 formulations significantly increase the relative abundance of microbiome functional pathways. HUMAnN3 was used to determine functional changes in the formulation-treated samples relative to Unt-Ctrl after 24 h of colonic stimulation. The top 20 regulated functional pathways ranked by effect size are shown for (**A**) AM-02 and (**B**) PM-02. Significance was measured between arms using LME. *P*-values for all tests were adjusted for multiple comparisons using the Benjamini-Hochberg method. *, *P* < 0.05; **, *P* < 0.01; ***, *P* < 0.001.

Metagenomic sequencing data were functionally profiled with HUMAnN3 to obtain relative abundances of annotated functional pathways mediated by the three formulations. The top 20 functional pathways significantly altered by AM-02 are reported (Fig. 4A), and AM-02 increased the relative abundance of multiple microbial functional pathways compared to Unt-Ctrl. Additionally, there was no significant decrease in any functional pathways by AM-02 treatment (Fig. 4A). In alignment with our data showing that AM-02 increases acetate and the abundance of acetate-producing microbes, AM-02 increased the PWY-5100 (pyruvate fermentation to acetate and lactate II) and P41-PWY (pyruvate fermentation to acetate and (S)-lactate I) pathways by 17% for both (*P* < 0.001), compared to Unt-Ctrl (Fig. 4A).

The top 20 functional pathways that were significantly regulated by PM-02 treatment highlight multiple tryptophan-producing pathways that are significantly increased in relative abundance compared to Unt-Ctrl (Fig. 4B). Specifically, the TRPSYN-PWY (L-tryptophan biosynthesis) was increased by 8% (*P* < 0.05), and the PWY-6629 (super-pathway of L-tryptophan biosynthesis) was increased by 6% (*P* < 0.05), both compared to Unt-Ctrl (Fig. 4B). Importantly, this PM-02-dependent increase in tryptophan-synthesis pathways coincided with an increase in the absolute abundance of tryptophan-producing bacteria, including species from the genera *Enterocloster* (530% increase, *P* < 0.01; Fig. S3B) and *Alloscillospira* (previously reported above as a SCFA-producing microbe) (Fig. 3C).

Because we saw a significant difference in functional pathways but not in the overall diversity of the microbiome at a taxonomic level, we hypothesized that the relative contribution to these pathways by individual bacterial species may be modified by response to the formulation treatments. For the PWY-5100 (pyruvate fermentation to acetate and lactate II) pathway, AM-02 treatment significantly increased the relative contribution of *Flavonifractor plautii, Coprococcus catus, Eubacterium ventriosum,* and *Bifidobacterium adolescentis,* while the contribution of *Collinsella aerofaciens* significantly decreased (all *P* < 0.05; Fig. S4A). For the P41-PWY (pyruvate fermentation to acetate and (S)-lactate I) pathway, AM-02 treatment significantly increased the contribution of *E. ventriosum* and *B. adolescentis*, whereas the contribution of *C. comes* and *C. aerofaciens* was significantly decreased (all *P* < 0.05; Fig. S4B). Furthermore, for the TRPSYN-PWY (L-tryptophan biosynthesis) pathway, PM-02 treatment significantly increased the contribution of *Escherichia coli, Odoribacter splanchnicus*, and *Blautia* sp. CAG 52 and significantly decreased the contribution of *Bacteroides vulgatus* (all *P* < 0.05; Fig. S4C). Finally, for PWY-6629 (superpathway of L-tryptophan biosynthesis), PM-02 treatment significantly increased the contribution of *E. coli* and *Anaerostipes hadrus*, while decreasing the contribution of *Ruminococcus torques* (all *P* < 0.05; Fig. S4D). Importantly, none of these bacterial species significantly changed in abundance, suggesting that this contribution to overall functional pathway abundances was a selective pressure for strains within these species that produce beneficial metabolites.

There were no pathways significantly altered by DM-02 treatment in this model compared to Unt-Ctrl. This suggests that either (i) effects of GABA in AM-02 and PM-02 formulations, but absent in DM-02, or (ii) synergy of specific ingredients within AM-02 and PM-02 formulations may be regulating the abundance of functional pathways and the contribution of specific bacterial species to the abundance of these pathways, in turn, affecting microbiome functional capacity and downstream metabolite production. This remains to be experimentally proven.

### Metabolite profile

**Fig 5:**
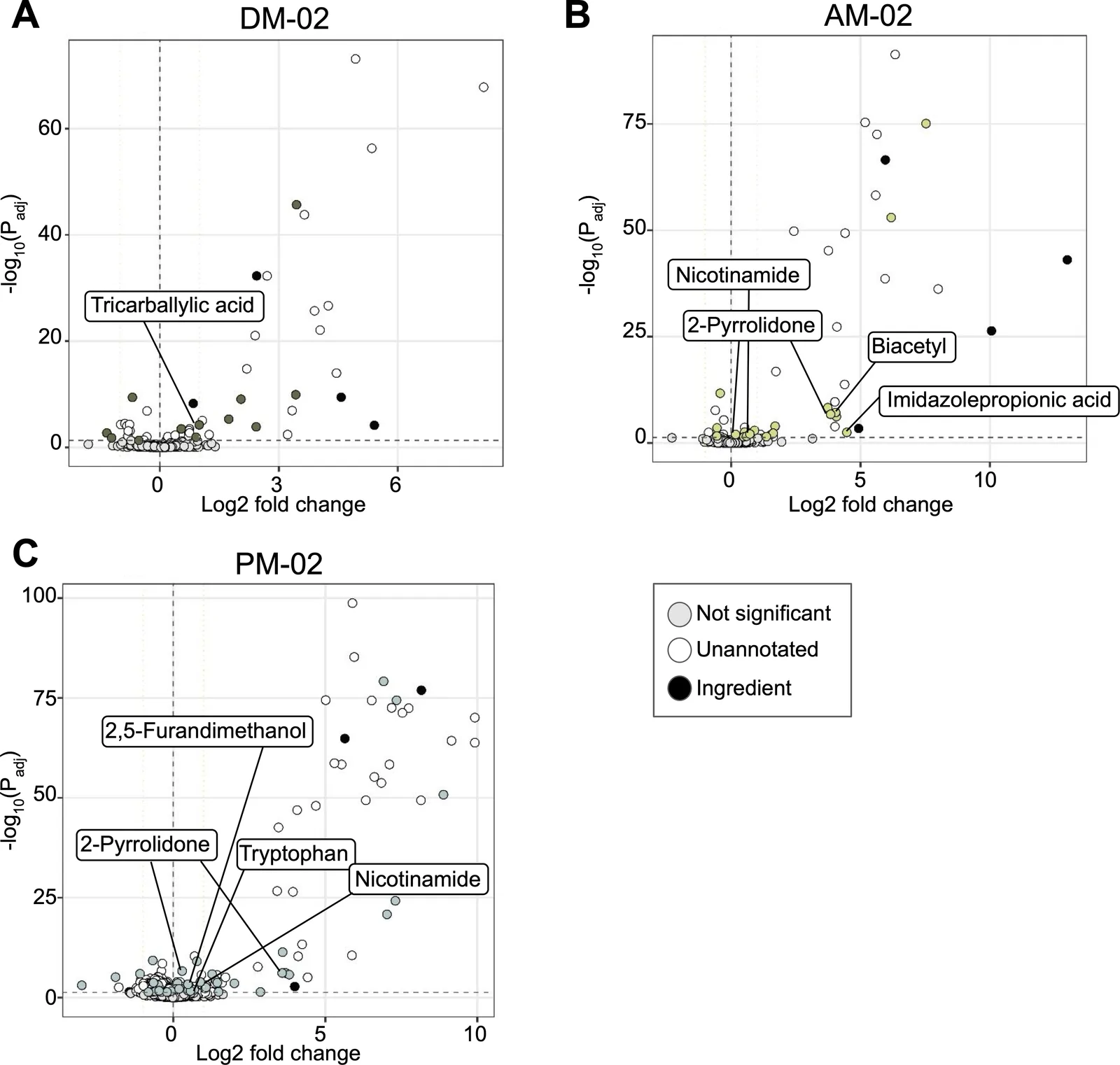
Formulations containing co-biotic compounds significantly impact the availability of metabolites. As described, the formulations underwent simulated digestion. Samples were then combined with healthy human stool from six individuals and entered colonic simulation for 24 h, with untargeted metabolomic output analyzed via LC-MS for (**A**) DM-02, (**B**) AM-02, and (**C**) PM-02, compared to Unt-Ctrl. Significance was measured between arms using LME. *P*-values for all tests were adjusted for multiple comparisons using the Benjamini-Hochberg method.

To assess the impact of formulation treatments on metabolite availability in this *ex vivo* GI model, untargeted metabolomics was applied to the post-colonic supernatants. The three formulations significantly altered the availability of many microbially derived metabolites from the post-colonic samples, compared to Unt-Ctrl (Fig. 5A through C; Table S3). Specifically, DM-02 treatment significantly increases the production of tricarballylic acid (*P* < 0.0001, 99% increase), a metabolite by-product of microbiome metabolism (Fig. 5A). This suggests that DM-02, containing co-biotics and high in vitamins and minerals, may alter microbial metabolism, potentially by increasing the availability of specific enzymatic co-factors (e.g., vitamin B1).

Both AM-02 and PM-02 treatment increased the availability of multiple annotated microbiome-regulated metabolites (Fig. 5B and C). In alignment with the functional pathway abundance data, a byproduct of pyruvate metabolism, biacetyl, increased by 1543%, compared to Unt-Ctrl (*P* < 0.0001; Fig. 5B). Furthermore, in the AM-02 treatment, 2-pyrrolidone (a cyclization product of GABA) (37) availability increased by 1,336% (*P* < 0.0001), imidazolepropionic acid (a histidine-derived gut microbial metabolite) availability increased 2,117% (*P* < 0.01), and nicotinamide (a vitamin B3 metabolite not found in the formulation) availability increased by 65% (*P* < 0.01) (Fig. 5B), all compared to Unt-Ctrl.

In agreement with our data showing PM-02 increases in tryptophan biosynthesis pathways and tryptophan-producing microbe data, PM-02 treatment increased tryptophan availability by 48% (*P* < 0.05), compared to Unt-Ctrl (Fig. 5C). Similar to the AM-02 treatment, PM-02 increased the availability of two forms of 2-pyrrolidone by 1,098% (*P* < 0.0001) and 22% (*P* < 0.0001) and nicotinamide by 85% (*P* < 0.001) (Fig. 5C). The availability of 2,5-furandimethanol (a byproduct of 5-hydroxymethylfurfural [HMF] reduction) was also increased by 40%, compared to Unt-Ctrl (*P* < 0.001). As a control, there were significant increases in some ingredients from the formulations (Table S3).

Together, these data suggest the ingredients within the co-biotic-containing formulations significantly impact the functional capacity and metabolic output of the microbiome of six donors.

## DISCUSSION

This mechanistic preclinical study showed that three novel formulations containing co-biotic compounds designed to support health significantly impact microbiome metabolic function and the composition of six healthy donor feces, while not increasing gas production in an *ex vivo* GI model. All three formulations decreased pH and increased SCFA production. Other functional and compositional changes in the microbiomes were largely formulation-dependent. These data suggest that the shared co-biotics (quercetin and vitamins B1, B2, and B3) found within the inner capsules of all formulations may drive overall beneficial microbial changes, and formulation-specific outer-capsule ingredients and their potential synergy with the co-biotic compounds determine finer tuning of the microbiome function and composition. Importantly, these results cannot determine the attribution of specific ingredients to phenotypes because these formulations are complex and contain formulation-specific ingredients. Thus, future mechanistic studies will be needed to elucidate which ingredients or combination of ingredients drive these microbiome outcomes. Additionally, the data from the six donor feces suggest that these novel formulations provide reproducible functional benefits across varied microbiomes in this model system.

The decreased colonic pH (by 3%–4%) with all formulation treatments was most likely driven by increased SCFA production (38, 39). This lowered pH has been shown to be beneficial to the host by driving increased mineral and electrolyte absorption in the colon and changes in the gut microbiota composition that inhibit growth of pathogens such as *Enterobacteriaceae* and *Clostridia* (40, 41). This mechanistic study does not show this causal relationship; thus, further studies are needed to confirm if this decrease in pH is sufficient to drive these two outcomes.

The three formulations increased acetate by 9%–12%, while AM-02 and PM-02 increased butyrate by 20% and propionate by 7%–8%, all of which have been shown to have a beneficial impact on the host microbiome and on human physiology via activation of G-protein coupled receptors, inhibition of histone deacetylases, and by serving as energy substrates (40). We acknowledge that in humans, the majority of SCFAs produced in the colon (~95%) are rapidly absorbed by the epithelial cells (40, 42–44); thus, we cannot directly correlate the increase of SCFAs in this study with human health. Clinical data would help contextualize these findings. Of note, the increases in total SCFA, acetate, butyrate, and propionate abundance by treatment with these formulations were consistent across all six donor feces, suggesting potential translation of these *in vitro* data to larger populations with diverse gut microbiomes (45).

Typically, during the fermentation of indigestible fibers, the increased production of SCFAs in the colon leads to an increase in the gases CO2 and H2 (40); however, this was not the case with the three tested formulations. The increase in SCFA and stable gas production seen in the DM-02 treatment arm may be due to the significant decrease in the abundance of the methane (CH₄) producer, *M. smithii*, which consumes H2 to produce methane as a byproduct. However, the method used to measure the accumulation of gases in the headspace does not distinguish between gas types; thus, confirming this mechanism will require follow-up experiments. This decrease in *M. smithii* was not seen in AM-02 and PM-02 treatments, but these treatments significantly increased butyrate production, and some cross-feeding of acetate to butyrate-producing microbes may be reducing gas production, although further studies are needed (34). These results suggest the three formulations containing co-biotics may have better tolerability than traditional fiber-heavy prebiotic formulations; however, this will require human studies for confirmation. This finding could inform the design of future clinical studies evaluating both efficacy and reproducibility in diverse human microbiomes.

There was an element of formulation-specific effects on the abundance of key beneficial microbes in this GI model system, although alpha- and beta-diversity were largely stable and donor-driven. This suggests that each formulation was modulating microbiome ecology at a species- or strain-level, but not at the overall diversity level, and in a formulation-dependent manner, which aligns with the differences in their outer-capsule ingredients. Although mostly different species were affected, each formulation increased the abundance of acetate- and butyrate-producing microbes, and the contribution of these SCFA-producing microbes to the total SCFA concentration will require follow-up experiments. Interestingly, each formulation increased the abundances of specific B-vitamin-producing microbes that may have an impact on both microbial and host cell function in the colon. Specifically, all three formulations increased species that produce vitamin B1 and B2, mentioned previously as enzymatic cofactors that drive both host and microbiome health. In addition, DM-02 increased the abundance of a microbe that produces vitamin B7 (biotin), a vitamin exclusively produced by microbes and plants that acts as a metabolic cofactor for microbes and human cells and promotes skin, nail, and hair health in humans (14, 46). Although biotin is largely absorbed in the proximal small intestine, it is also taken up via the colon from the diet or bacterial sources, and in animal models, the absence or depletion of the gut microbiome by antibiotics confirmed the microbial contribution to host biotin levels (47, 48). This is the first report of formulations containing co-biotic compounds promoting the growth of native microbes that produce co-biotic vitamins (vitamin B1 and B2) and essential vitamins (vitamin B7). Importantly, we have not determined that the abundance of these vitamin-producing microbes correlates with a physiologically relevant increase in the production of these vitamins in the colon; thus, determining this causative relationship will be a next step.

There were significant increases in both the abundances of functional pathways within the microbiomes treated with AM-02 and PM-02 and shifts in the contributions of specific species to these pathway abundances, collectively indicating functional changes in the microbiome. The increased abundance of two pyruvate to acetate/lactate pathways following AM-02 treatment may be the mechanism that drives the increase in acetate and butyrate production. Interestingly, AM-02 induced a selective pressure in this model system to increase the relative contribution of acetate/lactate pathways by known beneficial SCFA-producing species, *F. plautii, E. ventriosum*, and *B. adolescentis* (49–51), potentially by selecting for strains within these species that specifically contain these acetate-producing pathways. The extent to which this microbiome modulation by AM-02 translates to measurable physiological effects in humans remains to be explored.

Treatment with PM-02 increased the abundance of two tryptophan biosynthesis pathways and the abundance of two tryptophan-producing microbes, both of which may contribute to the increase in available tryptophan. As mentioned previously, microbiome-derived tryptophan promotes the synthesis of serotonin and melatonin from enterochromaffin cells within the gut epithelia to increase motility and visceral sensitivity, potentially contributing to the systemic pool of neurotransmitters that can regulate sleep quality and duration (52, 53). Further investigation into the impact of gut-derived serotonin and melatonin on neuronal function and pathways would be of interest. Notably, tryptophan can be utilized by the microbiome to promote the generation of neuroactive indole derivatives that signal via host receptors (e.g., AhR) to influence mucosal immunity and gut-brain communication (5, 54).

The ability of the formulations to modulate the metabolic output of the microbiomes was shown by the significant increase in the abundance of specific metabolites and vitamins. DM-02 specifically increased tricarballylic acid, indicating that treatment increases microbial tricarboxylate and citrate metabolism within the gut microbiome, which may equate to more favorable metabolic health profiles and modified microbiome-host interactions (55). Furthermore, niacin (vitamin B3 vitamer), a co-biotic compound found in the inner capsule of all three formulations, is the precursor utilized by both host and microbial cells to synthesize nicotinamide adenine dinucleotide (NAD) and subsequently nicotinamide (NAM, vitamin B3 vitamer), which was significantly increased in both AM-02 and PM-02 treatments. Because this GI model lacks host cells, the niacin-dependent increase in NAM reflects enhanced bacterial niacin utilization and NAD turnover (56). This turnover may be beneficial because higher microbial niacin/NAM use can support a more energetic, resilient microbiome and mediate vitamin B3-derived signals that help the host maintain NAD, gut barrier function, and mucosal homeostasis (57). Of note, these results are relative abundances based on untargeted metabolomics using Level 1 and 2 annotations, and future studies quantifying these metabolites compared to standards will be necessary to confirm these findings. Together, the formulation-specific impact on acetate and tryptophan pathways and associated metabolites supports the design of these formulations to fine-tune microbiome function, which may have an impact on host-microbiome communication and human health.

Although this study did not examine the co-biotic compounds on their own, it was interesting to note that AM-02 and PM-02 formulations with brain health-promoting GABA (58) were particularly effective at regulating the abundance of functional pathways and the contribution of specific bacterial species to the abundance of these pathways.

There are limitations to the experimental setup: this model lacks dynamic host functions of the gut, including epithelial cell nutrient and vitamin absorption and utilization in the colon, and does not capture immune responses. While the *ex vivo* SIFR technology enables controlled evaluation of microbiome functional responses independent of host variability, future clinical studies integrating host physiological endpoints will further validate and extend these findings. Importantly, while pathway enrichment and metabolite increases occur together, these data do not establish causal relationships. The study was set up not only to determine the co-biotic potential of each ingredient within the formulation but also to study only each formulation. To this point, these are complex formulations, and this study was not designed to look at microbiome regulation of specific ingredients. Additionally, the experiments only used one time point readout at 24 h; hence, extrapolation to later time points is not possible. Finally, as the six donors were healthy adults, the impact of these co-biotic formulations on individuals with metabolic, GI, or chronic conditions may differ.

While the purpose of this study was not to explore clinical benefits, it was notable that species, such as *F. plautii* (50), *E. ventriosum* (49, 59), and *B. adolescentis* (51), contributed to altered microbial function and are all promising health-promoting organisms. It is imperative that we confirm the contribution of these beneficial species to specific microbiome functions in humans to understand the impact of these formulations on human physiology.

In conclusion, the *ex vivo* GI model (SIFR technology) enabled integrated assessment of microbiome composition, metabolic activity, and metabolite production across six independent donor microbiomes under highly standardized conditions. This provided clinically relevant mechanistic insights into how novel co-biotic-containing formulations, engineered with a dual cap-in-cap delivery system, can enhance SCFA production, selectively enrich metabolite- and vitamin-producing taxa and pathways, and reshape post-colonic metabolite profiles, without increasing gas production. These findings provide a mechanistic foundation for clinical evaluation of co-biotic-containing formulations as targeted biotic interventions aimed at optimizing microbiome functional outputs while maintaining tolerability in diverse human populations.

## Data Availability

The data that support the findings of this study are openly available in Tables S2 and S3, the National Center for Biotechnology Information (NCBI) repository under accession no. PRJEB126105, and the MassIVE repository under accession number MSV000103203.

## SUPPLEMENTAL MATERIAL

Supplemental material aem.01311-26-s0001.pdf Fig. S1 to S4; Tables S1 to S3.

ASM does not own the copyrights to Supplemental Material that may be linked to, or accessed through, an article. The authors have granted ASM a non-exclusive, world-wide license to publish the Supplemental Material files. Please contact the corresponding author directly for reuse.
