# Supplementary material for: Formulations containing co-biotic compounds mediate microbiome function and composition without increased gas production in an ex vivo gastrointestinal model
Source: Appl Environ Microbiol. 2026 Oct 1;92(10):e01311-26. doi: 10.1128/aem.01311-26 (PMC13688805; doi:10.1128/aem.01311-26)
Supplement: Supplementary file 1 [file aem.01311-26-s0001.pdf]

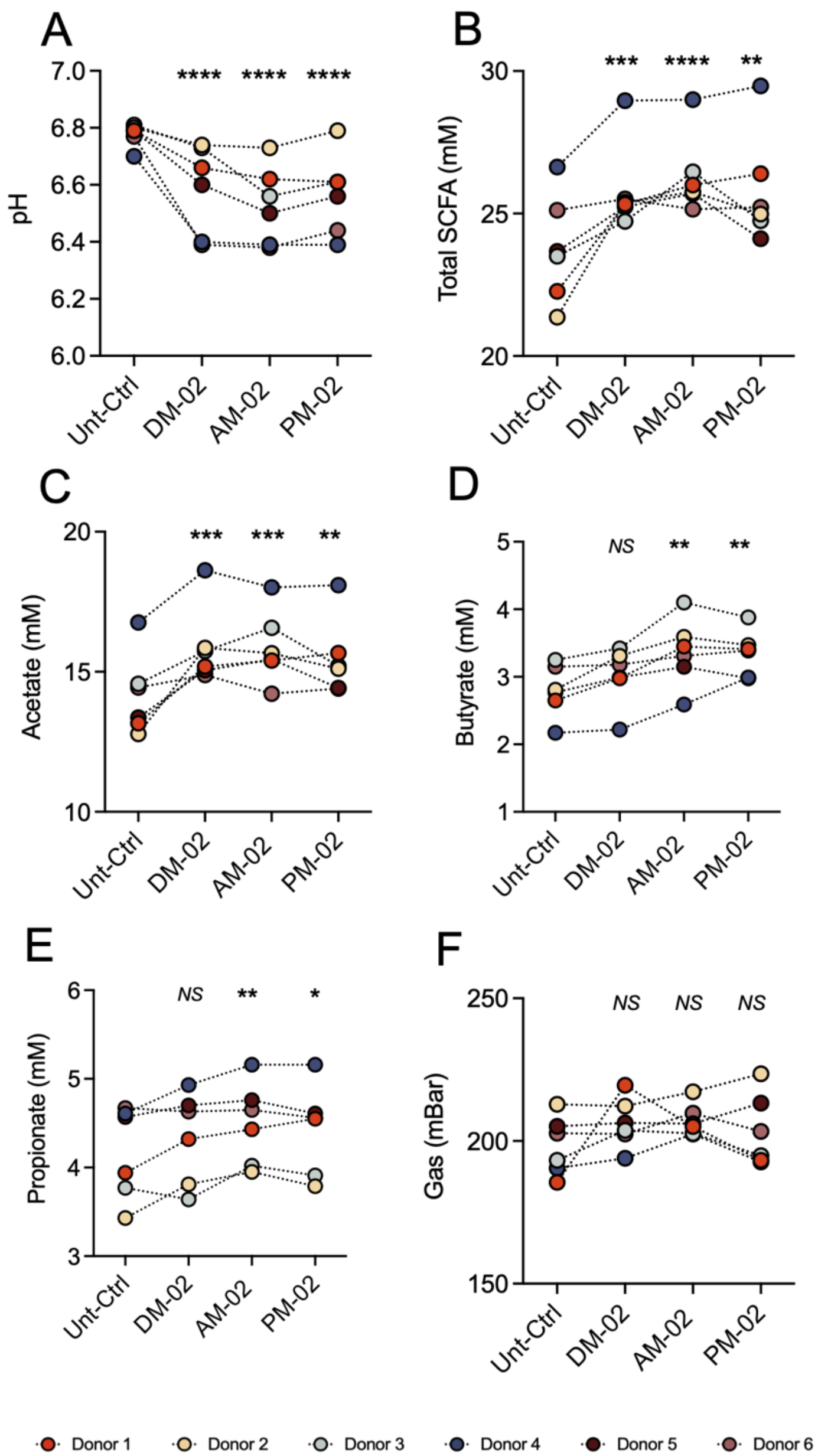

Supplemental Figure 1: Adapted results from Figure 2 shown as per-donor.

The same data depicted in Figure 2 of the main text but split on each donor (donors are separated by colors in the legend).

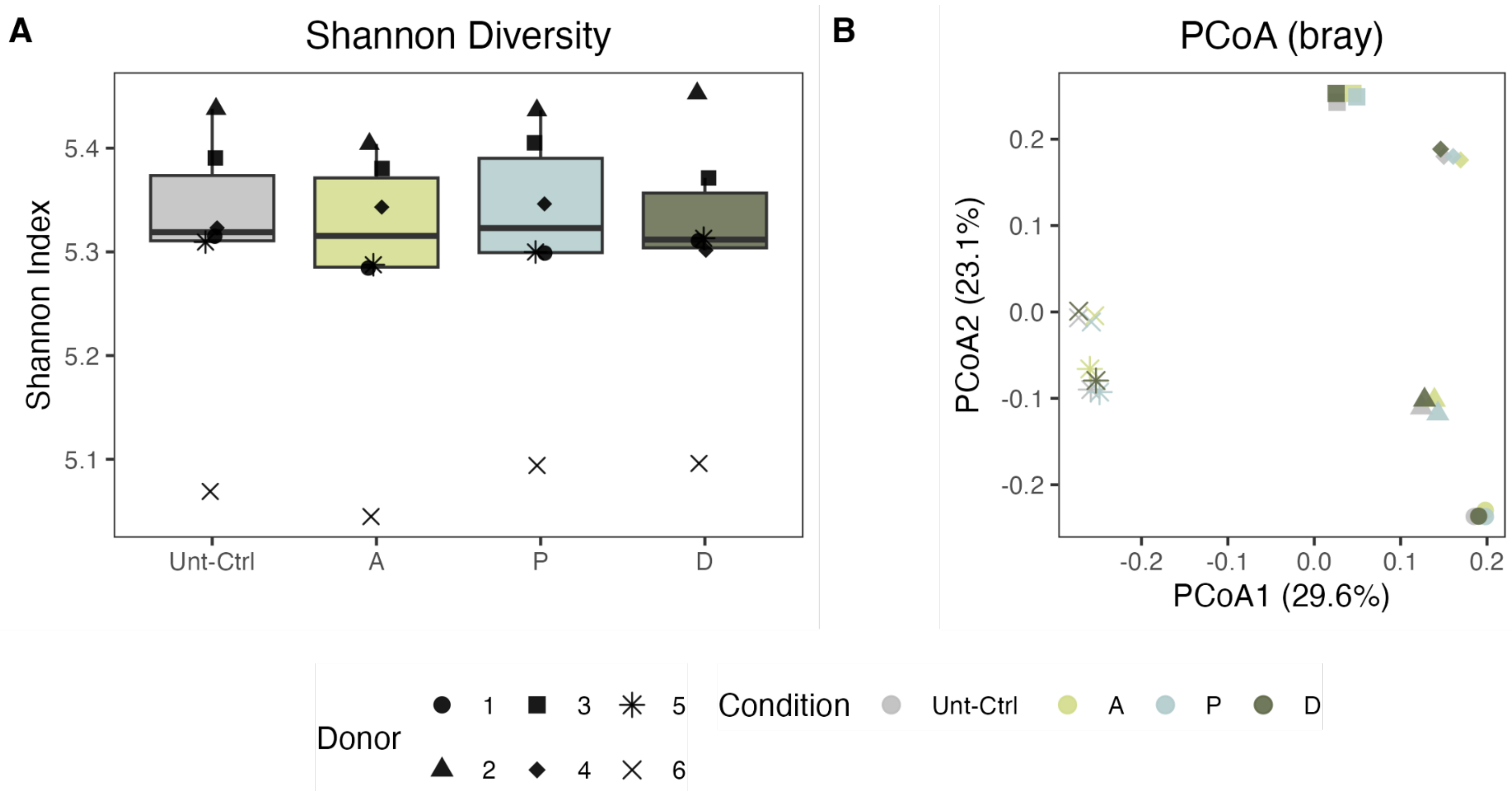

Supplemental Figure 2: The formulations did not significantly alter the overall microbiome diversity of the 6 health donors. A) Alpha-diversity via Shannon Diversity was measured between treatments and B) beta-diversity was measured via the Bray-Curtis distance matrix. Significance was measured between arms using linear mixed-effects modeling (LME). *p*-values for all tests were adjusted for multiple comparisons using the Benjamini-Hochberg method.

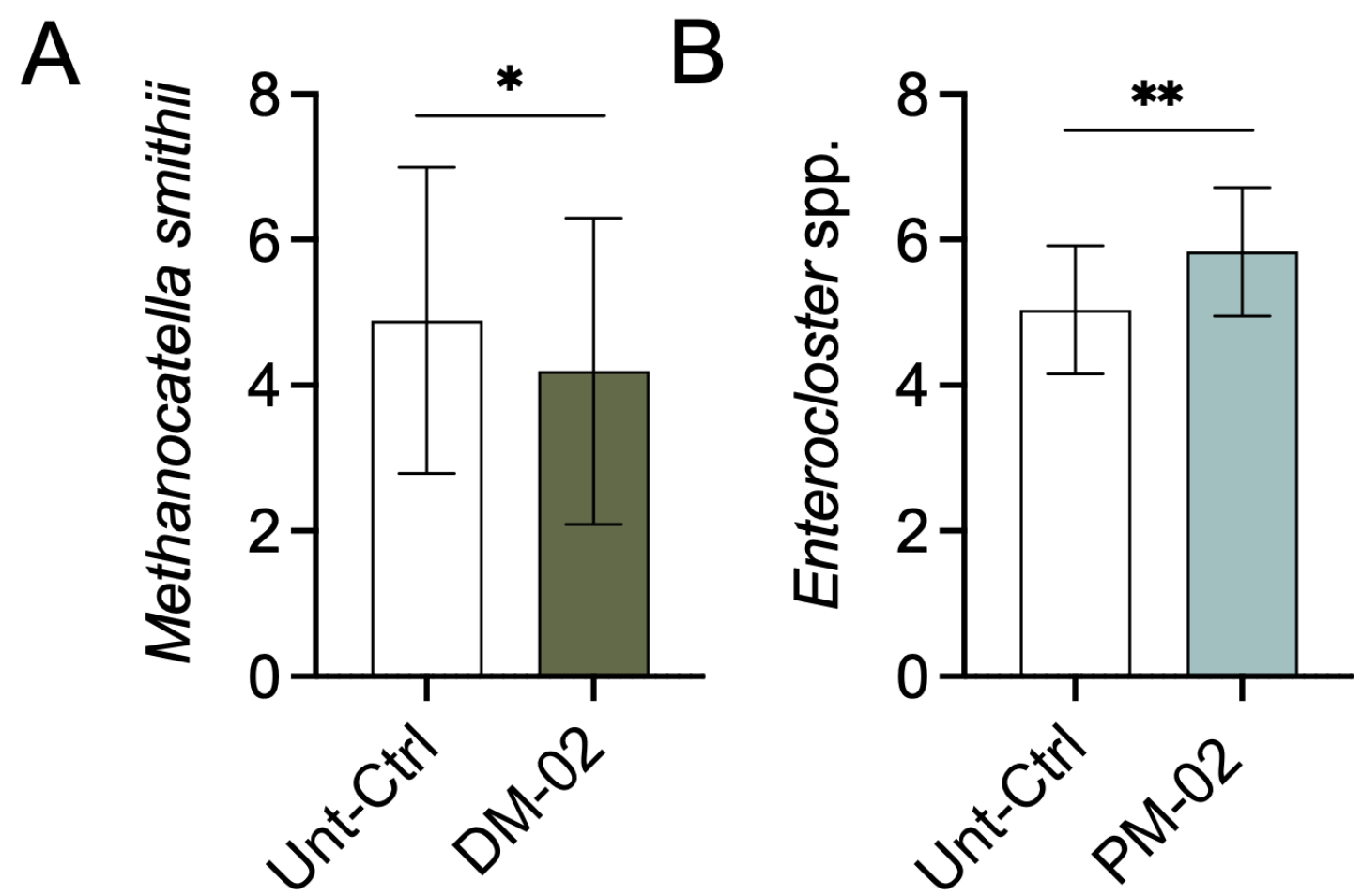

Supplemental Figure 3: The formulations containing co-biotics (DM-02, AM-02 and PM-02) alter the absolute abundance of key microbial species in the gut microbiome. A) DM-02 decreases the absolute abundance of a gas-producer, *Methanocatella smithii*, and B) PM-02 increases the absolute abundance of tryptophan-producing species from the genera *Enterocloster.* Significance was measured between arms using linear mixed-effects modeling (LME). *p*-values for all tests were adjusted for multiple comparisons using the Benjamini-Hochberg method.

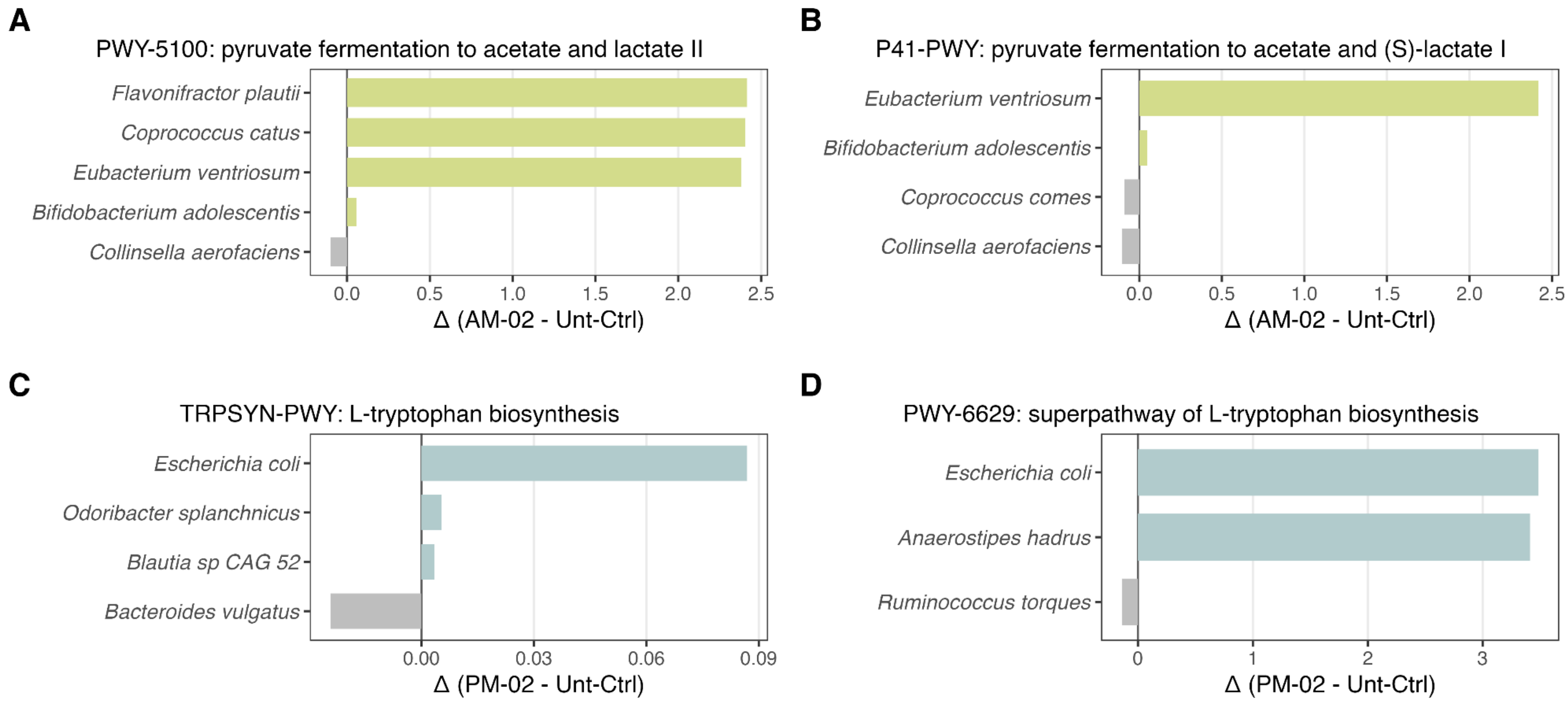

Supplemental Figure 4: The change in species contribution to the abundances of pyruvate fermentation to acetate and lactate and tryptophan functional pathways. For the selected pathways displayed in panels, a delta-matrix was constructed of relative abundance, log10 transformed species-level contributions in the A-B) AM-02 or C-D) PM-02 treatment groups relative to Unt-Ctrl. A two-sided *t*-test was used to assess whether the difference between the treatment and Unt-Ctrl differed from zero. Results with an unadjusted *p*-value threshold of ≤ 0.05 are displayed, and all unassigned taxa were omitted from plotting. The x-axis shows log10 fold change, and the color of the bar indicates the direction of enrichment.

Supplemental Table 1: Descriptions of the donors that provided a fecal donation for the current study.

#	Age	Sex	Race	BMI
1	35	Male	West-European	23.5
2	42	Female	West-European	19.9
3	48	Male	West-European	24.6
4	49	Female	West-European	19.2
5	27	Female	West-European	23.3
6	29	Male	West-European	21.9

Supplemental Table 2: All key fermentation parameters for six healthy donors.

Donor	Incubation time	Test condition ID	pH	Gas production [mbar]	Acetate [mM]	Propionate [mM]	Butyrate [mM]	Total_SCFA [mM]
1	24h	AM-02	6.62	205.07	15.40	4.43	3.45	26.00
2	24h	AM-03	6.73	217.22	15.66	3.95	3.59	25.76
3	24h	AM-04	6.56	202.64	16.57	4.02	4.10	26.46
4	24h	AM-05	6.39	202.42	18.01	5.16	2.59	29.00
5	24h	AM-06	6.50	206.07	15.44	4.76	3.15	25.70
6	24h	AM-07	6.38	209.71	14.22	4.65	3.31	25.15
1	24h	DM-02	6.66	219.51	15.19	4.32	2.98	25.33
2	24h	DM-02	6.74	212.21	15.85	3.81	3.31	25.39
3	24h	DM-02	6.73	203.69	15.74	3.64	3.42	24.73
4	24h	DM-02	6.40	193.95	18.62	4.93	2.22	28.96
5	24h	DM-02	6.60	206.34	15.05	4.70	2.99	25.24
6	24h	DM-02	6.39	202.47	14.88	4.63	3.18	25.51
1	24h	Unt-Ctrl	6.79	185.54	13.16	3.94	2.65	22.27
2	24h	Unt-Ctrl	6.80	212.87	12.78	3.43	2.81	21.36
3	24h	Unt-Ctrl	6.81	193.25	14.57	3.77	3.25	23.50
4	24h	Unt-Ctrl	6.70	190.45	16.76	4.61	2.17	26.63
5	24h	Unt-Ctrl	6.79	205.16	13.37	4.57	2.77	23.67
6	24h	Unt-Ctrl	6.77	202.71	14.44	4.67	3.15	25.12
1	24h	PM-02	6.61	193.22	15.67	4.55	3.41	26.40
2	24h	PM-02	6.79	223.58	15.11	3.79	3.47	24.98
3	24h	PM-02	6.61	194.86	15.21	3.91	3.88	24.75
4	24h	PM-02	6.39	192.65	18.09	5.16	2.99	29.48
5	24h	PM-02	6.56	213.30	14.42	4.61	2.98	24.12
6	24h	PM-02	6.44	203.36	14.40	4.56	3.39	25.22

<u>Supplemental Table 3</u>: All untargeted metabolites significantly increased by formulation treatment.

Treatment	Annotated Metabolite Name	p-value (adjusted)
AM-02	X04779_MB734	4.67E-92
PM-02	Melatonin	1.15E-77
AM-02	X03704_MB640	4.35E-76
AM-02	Lipoamide	7.99E-76
PM-02	X03704_MB640	3.49E-75
PM-02	Lipoamide	3.73E-75
PM-02	X04730_MB728	4.26E-75
DM-02	X03704_MB640	7.96E-74
AM-02	X04105_MB676	2.77E-73
PM-02	X02419_MB526	2.93E-73
PM-02	X01518_MB438	3.70E-73
PM-02	X01548_MB440	5.41E-72
PM-02	X00358_MB355	8.47E-71
DM-02	X01599_MB446	1.62E-68
AM-02	7-methylsulfinylheptyl isothiocyanate	2.80E-67
PM-02	7-methylsulfinylheptyl isothiocyanate	1.43E-65
PM-02	X00819_MB382	5.12E-65
PM-02	X00248_MB344	1.57E-64
PM-02	X05183_MB775	2.15E-59
PM-02	X01438_MB435	4.57E-59
PM-02	X03340_MB609	4.57E-59
AM-02	X03340_MB609	5.82E-59
DM-02	X03340_MB609	5.19E-57
PM-02	X02024_MB488	5.89E-56
PM-02	X01676_MB454	1.87E-54
AM-02	Protirelin	1.01E-53

PM-02	methyprylon	1.64E-51
AM-02	X03837_MB652	1.58E-50
PM-02	X03364_MB610	4.10E-50
PM-02	(22E)-3,5,9,14-Tetrahydroxyergosta-7,22-dien-6-one	4.21E-50
AM-02	X08319_MB1002	4.67E-50
PM-02	X01599_MB446	1.02E-48
PM-02	X08319_MB1002	1.17E-47
DM-02	Lipoamide	2.16E-46
AM-02	X06723_MB926	5.99E-46
DM-02	X06723_MB926	1.65E-44
AM-02	Theacrine, compound X00002	8.53E-44
PM-02	X06723_MB926	2.62E-43
AM-02	X02654_MB549	2.42E-39
AM-02	X00973_MB392	6.37E-37
DM-02	Pyridoxamine	5.43E-33
DM-02	X08319_MB1002	5.43E-33
AM-02	X04440_MB700	5.05E-28
PM-02	X04034_MB671	2.17E-27
DM-02	X04304_MB686	2.20E-27
PM-02	X04440_MB700	3.44E-27
AM-02	Theacrine, compound X00022	4.43E-27
DM-02	X04440_MB700	2.01E-26
PM-02	Theacrine, compound X00002	6.08E-25
DM-02	X04795_MB737	8.41E-23
DM-02	X00358_MB355	9.31E-22
PM-02	5-Allyl-5-sec-butyl-1,3-dimethyl-2,4,6(1H,3H,5H)-pyrimidinetrione	1.51E-21
AM-02	X01599_MB446	1.59E-17

DM-02	X00248_MB344	1.69E-15
DM-02	X00071_MB316	1.08E-14
AM-02	X00071_MB316	1.75E-14
PM-02	X00071_MB316	4.85E-14
AM-02	Cyclo(L-Phe-L-Pro)	2.08E-12
PM-02	PRACTOLOL	4.38E-12
PM-02	X00428_MB359	2.82E-11
PM-02	X02467_MB534	4.44E-11
PM-02	X00170_MB334	4.68E-11
DM-02	Phosphocholine	1.22E-10
AM-02	X00170_MB334	2.16E-10
DM-02	Pyridoxal	3.67E-10
DM-02	Pinoresinol dimethyl ether	3.95E-10
PM-02	Pinoresinol dimethyl ether	5.25E-10
PM-02	8-Hydroxyquinoline	8.41E-10
DM-02	methyprylon	8.61E-10
PM-02	X05558_MB816	3.10E-09
AM-02	Olean-12-ene-3,16,21,22,28-pentol	5.04E-09
DM-02	7-methylsulfinylheptyl isothiocyanate	5.47E-09
PM-02	X02739_MB558	2.10E-08
AM-02	Pinoresinol dimethyl ether	2.19E-08
AM-02	Biacetyl	6.94E-08
DM-02	X00170_MB334	1.19E-07
DM-02	X05558_MB816	1.40E-07
AM-02	2-Pyrrolidone, compound X00214	1.82E-07
PM-02	X01006_MB397	2.26E-07
PM-02	2-Pyrrolidone, compound X02139	2.30E-07
PM-02	Biacetyl	5.91E-07

AM-02	Furan	6.51E-07
PM-02	2-Pyrrolidone, compound X00214	7.32E-07
PM-02	Theacrine, compound X00022	8.30E-07
PM-02	Zalcitabine, compound X03602	1.12E-06
PM-02	Acetophenone	1.41E-06
PM-02	Furan	1.89E-06
PM-02	X01050_MB402	2.10E-06
PM-02	X02654_MB549	2.15E-06
AM-02	X05558_MB816	3.87E-06
DM-02	Choline	4.81E-06
PM-02	X02262_MB511	6.21E-06
PM-02	X03483_MB624	7.28E-06
PM-02	leucoline	7.79E-06
PM-02	X08503_MB1014	8.45E-06
PM-02	X01353_MB432	8.74E-06
DM-02	X00819_MB382	9.04E-06
PM-02	X02340_MB519	1.66E-05
PM-02	X05853_MB847	1.67E-05
PM-02	X06023_MB867	2.03E-05
PM-02	X08829_MB1034	2.26E-05
PM-02	X05454_MB802	2.65E-05
DM-02	X10806_MB1095	3.29E-05
PM-02	X05058_MB763	3.42E-05
PM-02	X01311_MB426	3.54E-05
PM-02	X08230_MB995	3.68E-05
PM-02	X05869_MB852	4.44E-05
PM-02	X04287_MB684	4.52E-05
DM-02	X11725_MB1115	4.78E-05

DM-02	X09201_MB1051	4.98E-05
PM-02	3-(3-Hydroxyphenyl)propanoic acid/ 3-(4-hydroxyphenyl)propionic acid/ 3-Phenyllactic acid	5.34E-05
PM-02	X09737_MB1068	5.34E-05
PM-02	X00999_MB396	5.48E-05
PM-02	X00573_MB368	5.78E-05
PM-02	X05445_MB801	5.78E-05
DM-02	Tricarballylic acid	5.86E-05
PM-02	X04884_MB747	6.16E-05
PM-02	X06170_MB883	6.76E-05
PM-02	X06420_MB905	6.92E-05
DM-02	Nicotinic acid	7.01E-05
PM-02	X03860_MB656	7.26E-05
PM-02	X04719_MB726	7.26E-05
PM-02	X05573_MB821	7.26E-05
PM-02	X02736_MB557	8.06E-05
PM-02	X02918_MB569	8.47E-05
DM-02	Biotin	8.96E-05
DM-02	X10900_MB1099	9.13E-05
PM-02	X03430_MB618	9.30E-05
PM-02	X01713_MB458	1.01E-04
PM-02	X05286_MB787	1.03E-04
PM-02	X00182_MB337	1.08E-04
PM-02	X05069_MB765	1.08E-04
PM-02	X03418_MB616	1.08E-04
PM-02	X01324_MB429	1.08E-04
PM-02	X05930_MB856	1.08E-04
PM-02	X08039_MB986	1.10E-04

PM-02	X01258_MB421	1.11E-04
AM-02	2-[4-(3-Hydroxypropyl)-2-methoxyphenoxy]-1,3-propanediol	1.15E-04
PM-02	X01906_MB477	1.16E-04
PM-02	X08191_MB991	1.18E-04
PM-02	X00205_MB342	1.20E-04
PM-02	X05475_MB805	1.20E-04
PM-02	X06061_MB871	1.20E-04
PM-02	Nicotine amide	1.32E-04
PM-02	X02097_MB497	1.32E-04
DM-02	Theacrine, compound X00002	1.36E-04
AM-02	X01353_MB432	1.41E-04
PM-02	X03933_MB664	1.47E-04
PM-02	X05140_MB769	1.47E-04
PM-02	(2R)-3-{[(2-Aminoethoxy)(hydroxy)phosphoryl]oxy}-2-[(1Z,11Z)-1,11-octadecadien-1-yloxy]propyl (13Z,16Z)-13,16-docosadienoate	1.47E-04
PM-02	X02931_MB572	1.53E-04
PM-02	X05390_MB793	1.59E-04
PM-02	X05862_MB850	1.60E-04
PM-02	X04899_MB749	1.74E-04
PM-02	X08440_MB1010	1.74E-04
PM-02	X00256_MB348	1.76E-04
PM-02	X05771_MB837	1.82E-04
PM-02	X03797_MB651	1.84E-04
PM-02	X02034_MB489	1.87E-04
PM-02	X02764_MB560	1.91E-04
PM-02	Procaine	1.96E-04

PM-02	X06887_MB935	1.96E-04
PM-02	2,4-Diamino-6-nitrotoluene	1.97E-04
PM-02	X09590_MB1064	1.97E-04
PM-02	X07246_MB954	1.98E-04
PM-02	X02928_MB571	2.01E-04
PM-02	X05760_MB834	2.03E-04
PM-02	X00973_MB392	2.08E-04
PM-02	X01257_MB420	2.10E-04
AM-02	X11077_MB1103	2.21E-04
PM-02	X08321_MB1003	2.24E-04
PM-02	X01582_MB444	2.27E-04
PM-02	X07540_MB965	2.39E-04
PM-02	Phosphoric acid	2.52E-04
PM-02	X06969_MB942	2.55E-04
PM-02	X07043_MB947	0.0002574122432
PM-02	X12342_MB1127	2.60E-04
PM-02	X10693_MB1089	2.67E-04
PM-02	X08694_MB1024	2.69E-04
PM-02	X03796_MB650	2.75E-04
PM-02	Creatinine	2.80E-04
PM-02	X12004_MB1119	2.80E-04
AM-02	(±)-1-Octen-3-ol	2.91E-04
PM-02	X08172_MB989	3.15E-04
PM-02	X09940_MB1076	3.19E-04
DM-02	Hydroquinone	3.32E-04
DM-02	2,5-Furandimethanol	3.32E-04
DM-02	X01050_MB402	3.32E-04
PM-02	X02971_MB580	3.36E-04

PM-02	X09803_MB1071	3.48E-04
PM-02	X07736_MB970	3.63E-04
PM-02	X02422_MB528	3.74E-04
PM-02	X11725_MB1115	3.85E-04
PM-02	X04697_MB723	3.90E-04
AM-02	Nicotinic acid	3.99E-04
PM-02	X08564_MB1021	4.06E-04
PM-02	X11039_MB1102	4.56E-04
PM-02	X07391_MB956	4.56E-04
PM-02	X10914_MB1101	4.56E-04
PM-02	Hydroquinone	4.91E-04
PM-02	2,5-Furandimethanol	4.91E-04
PM-02	X03625_MB632	4.91E-04
PM-02	X07881_MB975	4.91E-04
PM-02	X09297_MB1055	4.91E-04
PM-02	X09654_MB1066	5.14E-04
PM-02	X09063_MB1043	5.22E-04
PM-02	X08744_MB1029	5.41E-04
PM-02	X01040_MB400	5.97E-04
PM-02	X10063_MB1077	5.98E-04
PM-02	X01267_MB422	6.29E-04
PM-02	X05167_MB773	6.41E-04
PM-02	Pilocarpine, compound X02127	6.93E-04
DM-02	X01993_MB483	6.94E-04
PM-02	X03761_MB645	7.57E-04
PM-02	X05392_MB794	7.57E-04
PM-02	X00811_MB381	7.58E-04
DM-02	X10177_MB1081	7.73E-04

PM-02	X02187_MB505	7.96E-04
PM-02	X00254_MB347	8.02E-04
DM-02	X12111_MB1122	8.15E-04
PM-02	X01792_MB469	8.41E-04
PM-02	Bestatin	8.72E-04
PM-02	X01290_MB424	8.93E-04
PM-02	X03197_MB598	9.00E-04
PM-02	X08930_MB1039	9.09E-04
PM-02	X03427_MB617	9.15E-04
PM-02	X08143_MB988	0.00102878592
PM-02	X12196_MB1126	0.001125104621
AM-02	Gly-Trp	0.001131019976
PM-02	X14193_MB1144	0.001160823678
PM-02	X00502_MB365	0.001162115778
PM-02	phenacemide	0.001162115778
PM-02	X05249_MB782	0.001184608755
PM-02	X06081_MB874	0.001238300017
PM-02	X09524_MB1062	0.001325397557
PM-02	X01966_MB480	0.001487303539
PM-02	X08286_MB1001	0.001548751264
PM-02	X12111_MB1122	0.001561425123
PM-02	X10104_MB1078	0.001586574242
PM-02	X12400_MB1129	0.001586574242
PM-02	X12009_MB1120	0.001743159723
PM-02	Nicotinic acid	0.001767911041
PM-02	X10619_MB1087	0.001841446281
PM-02	X06222_MB887	0.001846788889
PM-02	X04585_MB711	0.00185268846

PM-02	X00954_MB390	0.001866589249
PM-02	X02386_MB523	0.001872773498
DM-02	Nornorcapsaicin	0.001959997973
DM-02	X05800_MB842	0.001959997973
PM-02	X03526_MB628	0.002090334885
PM-02	X02907_MB567	0.002150625918
PM-02	X03054_MB586	0.002150625918
PM-02	X00469_MB363	0.002293864233
PM-02	X04376_MB693	0.00239534265
PM-02	X10806_MB1095	0.002461329532
PM-02	(11R,23R)-14,17,20-Trihydroxy-23-(octanoyloxy)-14,20-dioxido-8,26-dioxo-9,13,15,19,21,25-hexaoxa-14λ~5~,20λ~5~-diphosphatritriacontan-11-yl undecanoate	0.002495589498
AM-02	[Similar to: 3-[5,7-dihydroxy-2-(4-hydroxyphenyl)-4-oxo-3,4-dihydro-2H-1-benzopyran-3-yl]-5,7-dihydroxy-2-(4-hydroxyphenyl)-3,4-dihydro-2H-1-benzopyran-4-one; ΔMass: -57.7700 Da], compound X07718	0.00272802954
PM-02	X01033_MB399	0.002748798829
PM-02	X03142_MB592	0.002808818007
PM-02	X09836_MB1073	0.002808818007
PM-02	X10177_MB1081	0.002808818007
PM-02	X05321_MB788	0.002887173189
PM-02	nitrosoproline	0.002910743867
PM-02	X03416_MB615	0.002966672594
PM-02	X01137_MB412	0.003268912504
AM-02	X08564_MB1021	0.003314609909
AM-02	Imidazolepropionic acid	0.003324301025
PM-02	X00359_MB356	0.003416295704

PM-02	X02049_MB491	0.003523725351
DM-02	X01548_MB440	0.003546552012
PM-02	X09201_MB1051	0.003570720954
PM-02	X05186_MB776	0.003660415018
PM-02	X02620_MB544	0.003679679076
PM-02	Setoclavine, compound X06391	0.003686106976
AM-02	X01050_MB402	0.003851753215
DM-02	X01353_MB432	0.003972428095
PM-02	X09110_MB1045	0.003988756493
DM-02	X08503_MB1014	0.004026073799
PM-02	X00211_MB343	0.004030656104
PM-02	X04779_MB734	0.004055056663
PM-02	X11895_MB1118	0.004412466319
PM-02	X00180_MB336	0.004555839582
PM-02	X02453_MB532	0.004591056065
PM-02	X06432_MB906	0.004620272271
PM-02	X10900_MB1099	0.004640563756
PM-02	X06390_MB901	0.004845675337
PM-02	X01329_MB430	0.004916244932
AM-02	PRACTOLOL	0.005136080459
DM-02	X08237_MB997	0.005196968471
PM-02	Imidazole-4-ylacetic acid/Imidazol-1-yl-acetic acid	0.005252064579
PM-02	X02769_MB562	0.005337399877
PM-02	X00603_MB371	0.005421794812
PM-02	X06027_MB868	0.005421794812
PM-02	X05750_MB833	0.005513075642
PM-02	Salicyluric acid	0.00558940783
PM-02	X00900_MB388	0.005746131728

PM-02	X01220_MB418	0.005746131728
PM-02	X04383_MB694	0.005746131728
PM-02	X10820_MB1096	0.005746131728
PM-02	X09182_MB1048	0.005874411562
PM-02	X01730_MB462	0.0059122146
PM-02	X06263_MB889	0.005983366625
PM-02	X01093_MB409	0.006321629856
PM-02	X00379_MB357	0.006388311092
PM-02	X00868_MB383	0.006468053164
PM-02	X07018_MB945	0.00656294974
PM-02	X06209_MB886	0.006603353863
PM-02	C22 Phytoceramide (t18:0/22:0)	0.006829503357
PM-02	Setoclavine, compound X10981	0.007034835047
PM-02	X09276_MB1053	0.007231681165
PM-02	Pyrophosphoric Acid	0.007233726192
DM-02	X08564_MB1021	0.007325814182
PM-02	X04524_MB706	0.007461370353
PM-02	X04605_MB715	0.007461370353
PM-02	X04816_MB738	0.007461370353
PM-02	X09397_MB1059	0.008137027241
AM-02	Nicotine amide	0.0081595331
PM-02	X13446_MB1138	0.00833502751
PM-02	X00092_MB324	0.00842175896
AM-02	X02649_MB547	0.008460351265
AM-02	X02202_MB507	0.008867200771
PM-02	X08377_MB1007	0.008941562088
PM-02	X01977_MB482	0.009476112951
DM-02	X05060_MB764	0.009619129327

AM-02	X04226_MB681	0.009736748944
AM-02	2-Pyrrolidone, compound X02139	0.0100824508
PM-02	X01673_MB452	0.01034434653
DM-02	X02769_MB562	0.01043108075
PM-02	X02418_MB525	0.01089138679
PM-02	X02454_MB533	0.01089542261
PM-02	X03840_MB654	0.01117555294
PM-02	X07587_MB967	0.01131475016
PM-02	X00446_MB360	0.01150122396
PM-02	Leu-pro, compound X00306	0.01154141059
PM-02	X07412_MB958	0.01178094238
PM-02	X08246_MB1000	0.01221188246
DM-02	(22E)-3,5,9,14-Tetrahydroxyergosta-7,22-dien-6-one	0.01221847053
PM-02	X03434_MB619	0.01258130903
AM-02	X07534_MB964	0.0128349225
PM-02	X01076_MB404	0.01337929293
PM-02	X00075_MB317	0.01356910658
PM-02	X00994_MB395	0.01356910658
PM-02	X01142_MB413	0.01356910658
PM-02	X08902_MB1038	0.01401883072
PM-02	X02202_MB507	0.01453945834
PM-02	X06373_MB900	0.01453945834
PM-02	X04328_MB690	0.01475244127
PM-02	X02835_MB564	0.01580187846
PM-02	X06164_MB881	0.01596718308
PM-02	X01164_MB415	0.01604076583
DM-02	leucoline	0.01616327934
PM-02	X06191_MB884	0.01618930395

PM-02	X02224_MB508	0.01644470322
PM-02	X02286_MB513	0.01736793167
PM-02	X06948_MB941	0.01753364701
PM-02	Methyl bisnorbiotinyl ketone	0.01905851189
PM-02	O-Ethyl (4-hydroxyphenyl)carbamothioate	0.01905851189
PM-02	Biotin	0.02014796086
PM-02	X03034_MB584	0.02075577222
PM-02	X01652_MB450	0.02088280356
PM-02	N(1),N(8)-bis(coumaroyl)spermidine	0.02112332505
PM-02	X01321_MB428	0.0212509109
PM-02	[Similar to: 3-[5,7-dihydroxy-2-(4-hydroxyphenyl)-4-oxo-3,4-dihydro-2H-1-benzopyran-3-yl]-5,7-dihydroxy-2-(4-hydroxyphenyl)-3,4-dihydro-2H-1-benzopyran-4-one; ΔMass: -57.7700 Da], compound X07718	0.0212509109
PM-02	Tryptophan	0.02241205686
PM-02	X02512_MB537	0.02241205686
PM-02	X07962_MB980	0.02305573421
AM-02	Biotin	0.0241441943
PM-02	3-(1-hydroxyethyl)-2,3,6,7,8,8a-hexahydropyrrolo[1,2-a]pyrazine-1,4-dione, compound X00696	0.0259610607
PM-02	X02602_MB543	0.0259610607
PM-02	X02677_MB554	0.0259610607
PM-02	X03442_MB620	0.02688869605
AM-02	[Similar to: 3-[5,7-dihydroxy-2-(4-hydroxyphenyl)-4-oxo-3,4-dihydro-2H-1-benzopyran-3-yl]-5,7-dihydroxy-2-(4-hydroxyphenyl)-3,4-dihydro-2H-1-benzopyran-4-one; ΔMass: -57.7700 Da], compound X09950	0.02725885295

PM-02	X06435_MB908	0.02748822806
PM-02	X01080_MB407	0.02904131183
PM-02	X02156_MB503	0.02942572511
PM-02	Hydroxycarteolol	0.03085145123
PM-02	X01077_MB405	0.03117747795
PM-02	X10905_MB1100	0.03121859684
PM-02	X11694_MB1114	0.03168012548
PM-02	X13840_MB1141	0.03171768111
PM-02	Indole-3-propionic acid	0.03252356791
PM-02	X02346_MB521	0.03255906398
PM-02	X07173_MB953	0.03277770513
AM-02	3-Methylhistidine	0.03331458841
AM-02	X08829_MB1034	0.03331458841
PM-02	X00874_MB384	0.03369774381
PM-02	3-Hydroxy-2-methylpyridine	0.03487688544
PM-02	γ-Aminobutyric acid/ 3-Aminoisobutanoic acid/ 2-Aminobutyric acid	0.03586934808
PM-02	X00577_MB369	0.03699339616
PM-02	Alanyltryptophan	0.03723816889
PM-02	X02532_MB539	0.03725538205
AM-02	2-Hydroxyfelbamate	0.03789945648
PM-02	X01452_MB436	0.03793147604
AM-02	X05714_MB829	0.03855756278
PM-02	X11077_MB1103	0.03912111419
AM-02	X00193_MB340	0.03926004653
PM-02	X14233_MB1145	0.04002528767
PM-02	Bicine/1-Deoxynojirimycin/ 1-Deoxymannojirimycin/ 1-Deoxymannojirimycin	0.04081914632

PM-02	X05556_MB815	0.04081914632
PM-02	5-Methyltryptophan	0.0411152096
PM-02	X02059_MB493	0.04141590539
PM-02	[Similar to: 3-[5,7-dihydroxy-2-(4-hydroxyphenyl)-4-oxo-3,4-dihydro-2H-1-benzopyran-3-yl]-5,7-dihydroxy-2-(4-hydroxyphenyl)-3,4-dihydro-2H-1-benzopyran-4-one; ΔMass: -57.7700 Da], compound X09950	0.04172147775
PM-02	4-Nitrobenzaldehyde	0.04178009565
PM-02	Dimethyl sulfoxide	0.04193039081
DM-02	X04247_MB683	0.04252159729
PM-02	X00802_MB379	0.04399590222
PM-02	X01247_MB419	0.04434970407
PM-02	8-Methyl-8-azabicyclo[3.2.1]octane-1,2,3,4,6-pentol	0.04434970407
PM-02	4-Hydroxyprolylleucine	0.04434970407
PM-02	Val-Ser, compound X05178	0.04434970407
PM-02	6-Acetamido-2-oxohexanoic acid	0.04434970407
PM-02	X05714_MB829	0.04461971969
PM-02	X01674_MB453	0.04523008659
PM-02	X13314_MB1136	0.04580447327
AM-02	X00248_MB344	0.04624044133
PM-02	X06478_MB914	0.04631567706
PM-02	X03657_MB637	0.04631681241
PM-02	X07165_MB952	0.04659745322
DM-02	X02083_MB495	0.04719279279
PM-02	X02126_MB500	0.04836654964
PM-02	X05880_MB853	0.04847246542
PM-02	X04838_MB740	0.04888871943

PM-02	Methylguanidine	0.04897759798
PM-02	N-Methylaspartic acid	0.04897759798
PM-02	X09282_MB1054	0.04897759798
DM-02	Indole-3-acetaldehyde	0.04921507481
AM-02	X07308_MB955	0.04971807437
PM-02	X07534_MB964	0.04985950336
